# Integrative Omics Reveals Glutamine Catabolism‐Driven Apoptotic Suppression in Monocytes upon Mechanical Unloading

**DOI:** 10.1002/advs.202500585

**Published:** 2025-08-18

**Authors:** Yi Ding, Fan Tong, Mingqiu Liu, Pin Yang, Jing Zeng, Yinghua Wei, Chunlin Li, Dong Li, Cheng Chang, Yangjun Zhang, Shaoqiong Yi, Fan Hu, Wenjie Shu, Lingqiang Zhang, Chun‐Ping Cui

**Affiliations:** ^1^ State Key Laboratory of Medical Proteomics Beijing Proteome Research Center National Center for Protein Sciences (Beijing) Beijing Institute of Lifeomics Beijing 102206 China; ^2^ School of Basic Medical Sciences Anhui Medical University Hefei Anhui 230022 China; ^3^ Bioinformatics Center of AMMS Beijing 100850 China; ^4^ Department of Endocrinology The Second Medical Center & National Clinical Research Center for Geriatric Diseases Chinese PLA General Hospital Beijing 100036 China; ^5^ School of Medicine Tsinghua University Beijing 100084 China

**Keywords:** bone resorption, glutamine, intrinsic apoptosis pathway, K63‐linkage ubiquitylation, mechanical unloading

## Abstract

Osteoclasts derived bone marrow monocytes have been documented to modulate bone quality by directly sensing mechanical forces. However, the mechanisms by which osteoclasts perceive and respond to mechanical disturbances remain unclear. Through integrating multi‐omics data of bone tissues from hindlimb unloading (HLU) and control mice, it is revealed that glutamine (Gln) catabolism‐induced suppression of apoptosis is critical for monocytes sensing and responding to mechanical unloading. Gln uptake is essential for the survival of monocytes under mechanical unloading. Deprivation of Gln or blockade of Gln transporter solute carrier family 1 member 5 (SLC1A5) inhibits bone resorption by enhancing apoptosis of monocytes. Unloading exposure‐induced cell survival is mediated by X‐linked inhibitor of apoptosis protein (XIAP)/direct IAP binding protein with low pI (Diablo) axis. Upon mechanical unloading XIAP is upregulated, then interacts with Diablo in mitochondrial and promotes the K63‐linkage ubiquitylation of Diablo at the K212 site. This sequesters Diablo within the mitochondrial and inhibits its release into the cytosol, ultimately inhibiting cell apoptosis of osteoclasts and the precursors. Clinically, the serum Gln levels are positively correlated with cross linked C‐telopeptide of type I collagen (CTX) levels, indicating that serum Gln levels might serve as a potential biomarker for predicting the risk of osteoporosis. Gln‐deficient diet, as well as SLC1A5 inhibitor L‐γ‐Glutamyl‐p‐nitroanilide (GPNA), effectively preserves bone mass in HLU mice, implicating attractive approaches for combating bone loss induced by weightlessness or disuse.

## Graphical abstract

This research integrated multi‐omics analysis of bone tissue from HLU and control mice revealed that mechanical unloading suppresses intrinsic apoptosis and augments glutamine (Gln) catabolism in osteoclast lineage cells. The findings highlight pivotal roles for SLC1A5‐mediated Gln metabolism and XIAP/Diablo axis‐mediated apoptosis suppression. And developed safe, potent bone‐loss therapeutics targeting Gln/SLC1A5.

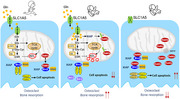

## 1 Introduction

Mechanical loading plays a crucial role in bone remodeling and maintaining bone health.^[^ ^1^, ^2^, ^3^, ^4^, ^5^, ^6^ ^]^ Osteoblast lineage cells have been documented to be capable of sensing and transmitting mechanical signals through a variety of pathways, including PIEZO channels, G‐protein coupled receptors, Wnt/β‐catenin and non‐coding RNAs.^[^ ^7^, ^8^, ^9^, ^10^, ^11^ ^]^ Osteoclasts had been reported to have the potential to directly sense mechanical forces.^[^ ^12^, ^13^, ^14^, ^15^ ^]^ Although calcium‐activated chloride channel Anoctamin‐1^[^ ^12^ ^]^ were identified to play an essential role in this process, there is very limited knowledge of the mechanisms by which osteoclasts directly sense and react to mechanical stress. Here, through integrating multi‐omics data (single‐cell transcriptome, metabolome, proteome and ubiquitinome) of bone tissues from hindlimb unloading (HLU) and control mice, we revealed that glutamine (Gln) catabolism‐induced suppression of intrinsic apoptosis pathway was critical for osteoclasts sensing and responding to mechanical unloading.

Osteoclasts are large multinucleated bone‐resorbing cells formed by the fusion of monocyte‐derived precursors that are thought to undergo apoptosis after a short lifespan of ≈2 weeks.^[^ ^13^ ^]^ In various forms of osteoporosis, individual osteoclasts are seemingly more “active” as they dig deeper resorption cavities, but it is not clear whether these observations reflect a change in cell vigor or in lifespan of osteoclasts.^[^ ^14^ ^]^ Additionally, mechanical unloading had been documented to promote RNAKL‐induced osteoclasts differentiation in vitro. Is the survival of osteoclasts and their precursor cells affected during unloading exposure? What is the regulatory mechanism? Here, we found that X‐linked inhibitor of apoptosis protein (XIAP)‐mediated ubiquitylation of direct IAP binding protein with low pI (Diablo) played a crucial role in unloading‐induced suppression of apoptosis in monocytes. XIAP is the most potent member of the inhibitor‐of‐apoptosis (IAP) family in terms of its ability to directly suppress caspase activity.^[^ ^16^, ^17^ ^]^ The mitochondrial protein Diablo promotes apoptosis through physical interaction with the caspase pocket in the third baculoviral IAP repeat (BIR3) domain of XIAP, resulting in the release of XIAP‐bound‐caspase 9.^[^ ^15^, ^16^, ^17^, ^18^ ^]^ Diablo/SMAC has been implicated in tumor development owing to its proapoptotic effects, and small molecule effectors have been developed as a cancer treatment.^[^ ^19^ ^]^ Here, we reveal previously unrecognized mechanism by which XIAP regulates the subcellular localization of Diablo to suppress cell apoptosis.

Gln is the most abundant non‐essential amino acid in the circulation and serves as a carbon and nitrogen donor for the production of biomolecules.^[^ ^20^ ^]^ Gln is involved in the regulation of redox homeostasis and become conditionally essential in hyper‐catabolic states and in tissues weakened by damage or stress.^[^ ^20^, ^21^, ^22^ ^]^ Although Gln catabolism had been reported to play roles in osteoclastogenesis,^[^ ^23^, ^24^ ^]^ the mechanisms of initiating Gln catabolism during bones resorption remain elusive. At present, we demonstrated that Gln uptake and catabolism are essential for the survival of monocytes during unloading‐induced bone loss. Gln influx and catabolism is dramatically elevated in the monocytes with unloading exposure. Deprivation of Gln or blockade of the Na^+^‐dependent Gln transporter solute carrier family 1 member 5 (SLC1A5)/alanine‐serine‐cysteine transporter 2 (ASCT2) significantly inhibited bone resorption by enhancing apoptosis of monocytes. Importantly, we demonstrated that targeting the Gln/SLC1A5 axis through SLC1A5 inhibitor L‐γ‐Glutamyl‐p‐nitroanilide (GPNA) or Gln‐deficient diet, effectively preserved bone mass in HLU mice, implicating attractive approaches for combatting bone loss induced by weightlessness or disuse.

## 2 Results

### 2.1 Integrative Proteomics Revealed Ubiquitylation Patterns in Bone Tissue upon Reduced Mechanical Stress

**Figure 1:**
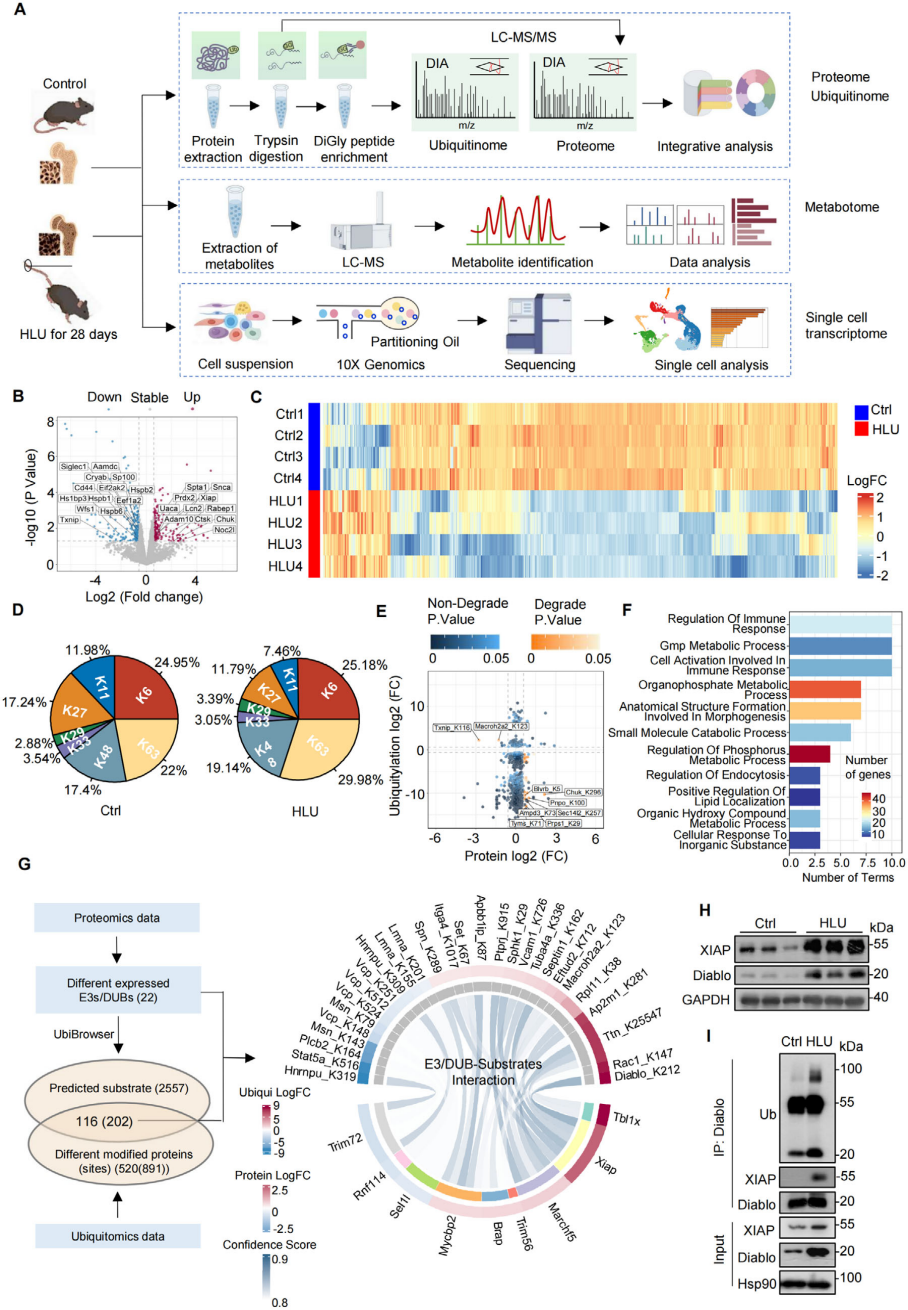
Integrative proteomics revealed ubiquitylation patterns in bone tissue upon reduced mechanical force. A) Schematic diagram of the procedure for integrative analysis of proteome, ubiquitinome, metabolome and single cell transcriptome of bone tissues from hindlimb unloading (HLU) and control (Ctrl) mice. Proteome and ubiquitinome identify stress‐responsive proteins and ubiquitination patterns under mechanical unloading. Single‐cell transcriptome analysis resolves cell‐type‐specific gene expression alterations and functional enrichment. Metabolomic analysis quantifies mechanical stress‐induced shifts in amino acid metabolism. Integration of these multi‐omics layers reveals mutually reinforcing biological evidence. B) The volcano plot displayed differentially expressed proteins (DEP) between bone tissues from HLU and Ctrl mice. The apoptosis‐related proteins are highlighted. Red: upregulated proteins; Blue: down‐regulated proteins. *n* = 4 per group. C) Heatmap showing the scaled expression of differentially expressed ubiquitination sites in eight samples. Red: high expression; Blue: low expression. *n* = 4 per group. D) Pie charts showing the coverage of different ubiquitin chains for each group. *n* = 4 per group. E) Scatter plot of degradative and non‐degradative ubiquitylation. Degradative ubiquitylation sites were labeled. Red: degrade; Blue: non‐degrade. *n* = 4 per group. F) Enrichminer was employed to perform functional enrichment analysis on the non‐degradative ubiquitylation proteins, and the resulting representative terms were visualized in a histogram. *n* = 4 per group. G) Integrative analysis framework for proteome and ubiquitinome. The chordal graph (right) displays the relationship between E3 ubiquitin ligase in the proteome data and their corresponding predicted substrates in the ubiquitinome data. The depth of color in the lines indicates the predicted confidence score, while the outer ring's color represents the log2 fold change (log2FC) of the corresponding proteins. *n* = 4 per group. H) Immunoblot analysis of XIAP and Diablo in bone tissue from HLU or Ctrl mice. *n* = 3 per group. I) The lysates of bone tissue from HLU and Ctrl mice were subjected to immunoprecipitation (IP) and immunoblot with indicated antibodies. *n* = 3 per group.

To map the biogeography of ubiquitin modification in bone tissue in response to mechanical stress, we conducted proteome and ubiquitinome profiling on the bone tissue samples from HLU and control mice (**Figure** **1**A; Figure S1A,B, Supporting Information). Whole cell proteome (WCP) analysis was performed to quantify and compare protein abundance. We identified 5114 proteins in at least three of the four experiments, and 4718 proteins (71.76% of total proteins) were identified in all the 8 samples (Figure S1C, Supporting Information). Spearman correlation coefficients ranged between 0.91 and 0.97 for pair‐wise comparisons of the normalized and log2‐scaled protein abundances in control and HLU samples (Figure S1D, Supporting Information). Principal component analysis (PCA) showed the difference between HLU and control samples (Figure S1E, Supporting Information). Compared with the control, 196 proteins were increased, and 217 proteins were decreased in the bone tissues from HLU mice (Figure 1B). The KEGG pathway analysis revealed an enrichment of apoptosis, lysosome and ferroptosis‐related pathways among the up‐regulated proteins (Figure S1F, Supporting Information), while focal adhesion and cell junction were found to be enriched among the down‐regulated proteins (Figure S1G, Supporting Information).

We employed di‐glycine (diGly) antibody‐based enrichment and data‐independent acquisition (DIA) method for ubiquitinome analysis,^[^ ^25^, ^26^ ^]^ and successfully quantified a total of 6501 non‐redundant diGly‐modified sites in 2210 proteins (Figure S1H, Supporting Information). Spearman's correlation coefficients between 0.67 and 0.94 indicated high reproducibility between biological replicate samples (Figure S1I, Supporting Information). PCA showed the significant difference between bone tissues from control and HLU mice in ubiquitinome (Figure S1J, Supporting Information). We quantified changes in the ubiquitylation of 520 proteins in HLU mice compared with the controls, and 117 diGly‐modified sites in 117 peptides were increased, and 774 sites in 773 peptides were decreased (Figure 1C). In addition, we analyzed the frequency of the number of ubiquitination sites per protein and observed that substrates were only generally ubiquitinated a few times on average (Figure S1K, Supporting Information). However, there are outliers, with some proteins being extensively ubiquitinated, although the biological significance of this is not yet clear. The most frequently 10 ubiquitinated domains (myosin motor, protein kinase, USP, etc) were highlighted (Figure S1L, Supporting Information). Decreased diGly‐modified sites primarily are in the domains of Protein Kinase, suggesting that ubiquitination in response to mechanical unloading might be linked to phosphorylation (Figure S1M, Supporting Information). Subsequently, we attempted to assess the average change in relative abundance of the ubiquitin lysine di‐Gly peptides. We found that K63 peptides were increased, while K11 and K27 peptides were decreased in HLU bone tissues compared with the controls (Figure 1D). The abundance of K48 ubiquitin remnant peptides, associated with proteasomal degradation, remain unchanged (Figure 1D).

We next integrated the WCP and ubiquitin remnant datasets to specifically interrogate patterns for protein ubiquitylation in bone tissue upon mechanical unloading. Comparison between di‐Gly profiling and WCP predicted degradative ubiquitylation of 27 sites in 20 proteins and non‐degradative modification of 864 sites in 500 proteins (Figure 1E), suggesting that unloading exposure led to a significant increase in non‐degradative ubiquitylation. We used EnrichMiner,^[^ ^27^ ^]^ a complete pipeline for functional enrichment analysis and representative terms selection (https://omicsmining.ncpsb.org.cn/ExpressVis/EnrichMiner) to mine biological insight in degradative and non‐degradative ubiquitylation respectively. Eleven terms, such as small molecule metabolic process, establishment of protein localization, are enriched in the proteins with degradative ubiquitylation (Table S1, Supporting Information). 128 terms, such as myeloid cell activation involved in immune response, ubiquitin dependent endoplasmic reticulum associated degradation (ERAD) pathway, are enriched in the proteins with non‐degradative ubiquitylation (Figure 1F; Table S2, Supporting Information). Additionally, we mapped an ubiquitylation‐relevant protein–protein interaction (PPI) network between E3/DUB and substrates through comprehensive analysis of differential expressed ubiquitin enzymes (Figure S1N, Supporting Information) and ESI (E3‐substate interaction)/DSI (DUB‐substrate interaction) libraries of UbiBrowser.^[^ ^28^, ^29^, ^30^ ^]^ We identified a total of 282 pairs ESIs/DSIs, of which 55 pairs were retained based on the modifying relationship. The confidence score of the interaction between XIAP and Diablo ranked first among the retained ESIs/DSIs (Figure 1G). The levels of XIAP protein and Diablo ubiquitylation at the K212 site were up‐regulated by 5.6‐fold and 407‐fold respectively in the HLU bone tissues. Consistently, we observed that XIAP level was increased in the bone tissue of HLU mice (Figure 1H; Figure S1O, Supporting Information), whereas no significant enhancement in ovariectomized (OVX) mice (Figure S1O, Supporting Information). The ubiquitination modification of Diablo, as well as the interaction between XIAP and Diablo exhibited substantial augment in the bone tissue of HLU mice (Figure 1I). These findings suggested that XIAP‐regulated ubiquitylation of Diablo may play a role in unloading‐induced bone loss.

### 2.2 Mechanical Unloading Suppressed Intrinsic Apoptotic Pathway in Mononuclear Cells

**Figure 2:**
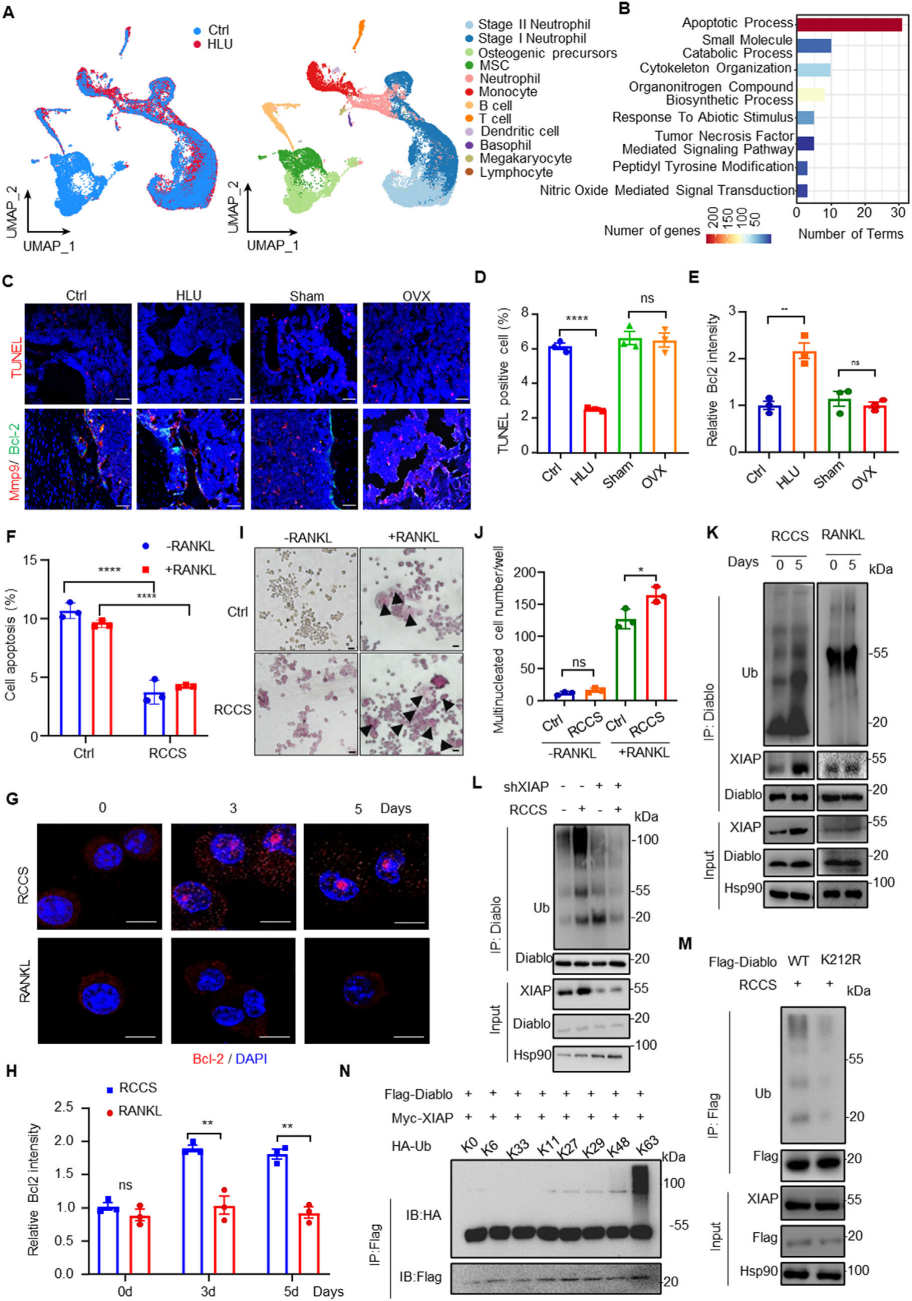
Mechanical unloading suppressed intrinsic apoptotic pathway in osteoclast lineage cells through XIAP‐mediated K63‐linkage ubiquitylation of Diablo. A) Single cell RNA (scRNA)‐Seq data was analyzed and cell clusters were graphically displayed by Uniform Manifold Approximation and Projection (UMAP) plot colored by sample groups (left) and cell type identifications (right). *n* = 2 per group. B) Enrichminer was employed to perform functional enrichment analysis on the differentially expressed genes (DEGs) of monocyte cluster in scRNA‐Seq data, and the resulting representative terms were visualized in a histogram. *n* = 2 per group. C) Immunofluorescence staining of Mmp9, Bcl‐2 and TUNEL in femurs from mice. Scale bars, 50 µm. D,E) Quantitative analysis was performed by ImageJ. *n* = 3 per group. F) RAW264.7 cells were cultured using either the RCCS or a normal culture system for 3 d. Subsequently, the cells were treated with 50 ng mL^−1^ RANKL and 30 ng mL^−1^ M‐CSF for 5 d or untreated. Annexin V assay was conducted to examine the percentage of apoptotic cells. *n* = 3 per group. G) Immunofluorescence staining of Bcl‐2 in RAW264.7 cells with RCCS or RANKL treatment. The nuclei were stained with DAPI. Scale bars, 10 µ. H) Quantitative analysis was by ImageJ. *n* = 3 per group. I) After Rotary Cell Culture System (RCCS) treatment or control treatment for 3 d followed by culture for 5 d with 30 ng mL^−1^ M‐CSF and 50 ng mL^−1^ RANKL, representative images of TRAP staining in RAW264.7 cells were shown and J) the numbers of multinucleated TRAP‐positive cells per well were quantified. Scale bars: 10 µm. *n* = 3 per group. K) Cell lysates were collected from RAW264.7 cultured with RCCS or RANKL. Immunoprecipitates of anti‐Diablo were subjected to immunoblot analysis. *n* = 3 per group. L) RAW264.7 cells with stable XIAP silence by shRNA were cultured with RCCS for 5 d, and the cell lysates were collected for Co‐IP assay and immunoblot analysis. *n* = 3 per group. M) The Diablo‐silenced RAW264.7 cells were stably transfected with Diablo wild type (WT) or K212R mutant and the cell lysates were collected for IP and immunoblot analysis. *n* = 3 per group. N) Diablo ubiquitylation linkage assay. HA‐ubiquitin WT or indicated mutants were co‐transfected with Flag‐Diablo and Myc‐XIAP in HEK293T cells. Cell lysates were collected for IP and immunoblot analysis. *n* = 3 per group. *P < 0.05, **P<0.01, ***P < 0.001, ****P < 0.0001, n.s., not significant. Data are mean ± SEM. Statistical differences were determined using Student's t‐test or ANOVA.

To explore whether different cell types in bone tissue deploy different patterns to cope with the lack of mechanical force, we performed single‐cell RNA sequencing (scRNA‐Seq) on the tibial and femoral tissue samples from HLU and control mice (Figure 1A; Figure S1A,B, Supporting Information). Unsupervised clustering identified 12 unique clusters across 2 control and 2 HLU samples (**Figure** **2**A), and differential expressed gene analysis indicated that each cluster had a unique gene signature (Figure S2A,B, Supporting Information). We observed a significant decrease in the cell count of osteoblast lineage cells (mesenchymal stem cells and pre‐osteoblast clusters) and a dramatic increase in monocytes (Figure 2A; Figure S2C, Supporting Information). To elucidate the underlying mechanism behind the increase of monocytes count upon unloading exposure, we employed Enrichminer to analyze the differentially expressed genes (DEGs) of monocytes (Figure S2D, Supporting Information) and found that cell apoptosis exhibits the most significant enrichment in terms of biological processes (Figure 2B). Total of 31 terms, including regulation of intrinsic apoptotic pathway (Table S3, Supporting Information), were enriched in the cluster of apoptotic process. The expression of multiple intrinsic apoptosis inhibition genes, such as members of the B‐cell lymphoma‐2 (Bcl‐2) family, is upregulated in monocytes derived from bone tissue of HLU mice, while pro‐apoptotic genes like BCL‐2‐associated X protein (Bax) and BCL2‐antagonist/killer (Bak) etc., are down‐regulated (Figure S2D, Supporting Information). To validate this signature in monocytes, we generated HLU and OVX‐induced osteoporosis mice model (Figure S2E–K, Supporting Information). We observed that terminal deoxynucleotidyl transferase mediated dUTP nick‐End labeling (TUNEL) positive cells were significantly decrease, and Bcl‐2 level was remarkedly increased in osteoclast lineage cells indicated by Mmp9 positive staining in HLU bone tissues compared with the controls (Figure 2C–E). However, these phenomena were not observed in the bone tissues from OVX mice (Figure 2C–E). Cell apoptosis in BMDM (bone marrow derived monocytes) was markedly decreased in HLU mice compared with the controls (Figure S3A, Supporting Information). Additionally, we utilized the rotary cell culture system (RCCS) developed by National Aeronautics and Space Administration to mimic mechanical unloading in vitro (Figure S3B, Supporting Information). The in vitro model of receptor activator of nuclear factor‐κ B ligand (RANKL)‐stimulated osteoclast differentiation was established and assessed (Figure S3C, Supporting Information). Cell apoptosis and Caspase‐3 cleavage were dramatically suppressed in RAW264.7 cells with RCCS culture (Figure 2F–H; Figure S3D, Supporting Information), suggesting that reduced mechanical loading might suppress intrinsic apoptotic pathway in mononuclear cells. Considering that RANKL is required for osteoclasts differentiation, we established an in vitro model combining RCCS with RANKL treatment to determine the effect of RCCS on osteoclasts differentiation. Briefly, Raw264.7 cells were treated with RCCS for 16 rpm for 3 d, followed by culturing cells for 5 d with RANKL. Intriguingly, RCCS culture significantly suppressed cell apoptosis in Raw264.7 cells either with or without RANKL treatment (Figure 2F). Multinucleated TRAP‐positive cells were observed in RANKL‐treated cells (Figure 2I,J), indicating that we successfully established an in vitro model for RANKL‐induced osteoclast differentiation. Almost no multinucleated TRAP‐positive cells were detected in RCCS‐treated group (Figure 2I,J; Figure S3E, Supporting Information), suggesting that RCCS treatment alone hardly induced the differentiation of Raw264.7 cells into osteoclasts. Intriguingly, the RCCS+RANKL‐treated group exhibited a higher number of multinucleated tartrate‐resistant acid phosphatase (TRAP)‐positive cells compared to the RANKL‐treated group, indicating that RCCS treatment promoted RANKL‐induced osteoclast differentiation (Figure 2I,J; Figure S3E, Supporting Information).

Considering the roles of XIAP and Diablo in the regulation of cell intrinsic apoptosis pathway, we proposed that XIAP‐mediated Diablo ubiquitylation might contribute to the suppression of monocytes apoptosis during mechanical unloading. RCCS culture or HLU dramatically increased the levels of XIAP mRNA and protein in Raw264.7 and BMDM cells (Figure S3F–H, Supporting Information), but not in osteoblast lineage cells (BMSC and MC3T3‐E1 cells) (Figure S3I,J, Supporting Information). We generated stable XIAP knockdown or overexpression Raw264.7 cells, and observed that the protein stability of Diablo is not been changed by XIAP knockdown or overexpression (Figure S3K,L, Supporting Information). The ubiquitylation modification of Diablo, as well as the interaction between XIAP and Diablo exhibited substantial enhancement in Raw264.7 cells cultured with RCCS (Figure 2K). Unlike RCCS culture, RANKL stimulation did not change XIAP expression, Diablo ubiquitylation and the interaction between XIAP and Diablo in Raw264.7 cells (Figure 2K; Figure S3F, Supporting Information), implying distinct mechanisms underlying bone resorption caused by RANKL and mechanical unloading.

### 2.3 XIAP Promoted K63‐Linked Ubiquitylation of Diablo and Sequestered It in Mitochondrial in Response to Unloading Exposure

In some in vivo and in vitro systems, XIAP was observed to induce ubiquitylation and proteasome‐dependent degradation of Diablo/SMAC via its E3 ligase activity.^[^ ^31^, ^32^, ^33^ ^]^ Whereas the other research showed that Diablo/SMAC can inhibit the autoubiquitylation of XIAP,^[^ ^34^ ^]^ and XIAP cannot ubiquitylate SMAC/Diablo.^[^ ^34^ ^]^ These disagreements may due to different cellular contexts and some currently unknown regulatory mechanisms. To determine if Diablo is the ubiquitylation substrate of XIAP, in vivo and in vitro ubiquitylation assays were performed. In vitro ubiquitylation assay showed that XIAP, but not the catalytically inactive H466A mutant, attached ubiquitin onto Diablo in cell‐free system (Figure S3M, Supporting Information). XIAP deficiency by shRNA suppressed the ubiquitin conjugation on Diablo upon unloading exposure (Figure 2L). The Diablo K212R mutant showed a dramatic decrease in the formation of ubiquitin conjugates in the cells with RCCS culture (Figure 2M) or XIAP overexpression (Figure S3N, Supporting Information), suggesting that K212 could be the major site for ubiquitylation of Diablo. Additionally, we ectopically expressed a series of ubiquitin mutants and performed a thorough ubiquitylation assay of Diablo by XIAP in cellular system. Diablo could be linked with only K63‐linkage ubiquitin in the presence of XIAP (Figure 2N; Figure S3O, Supporting Information).

**Figure 3:**
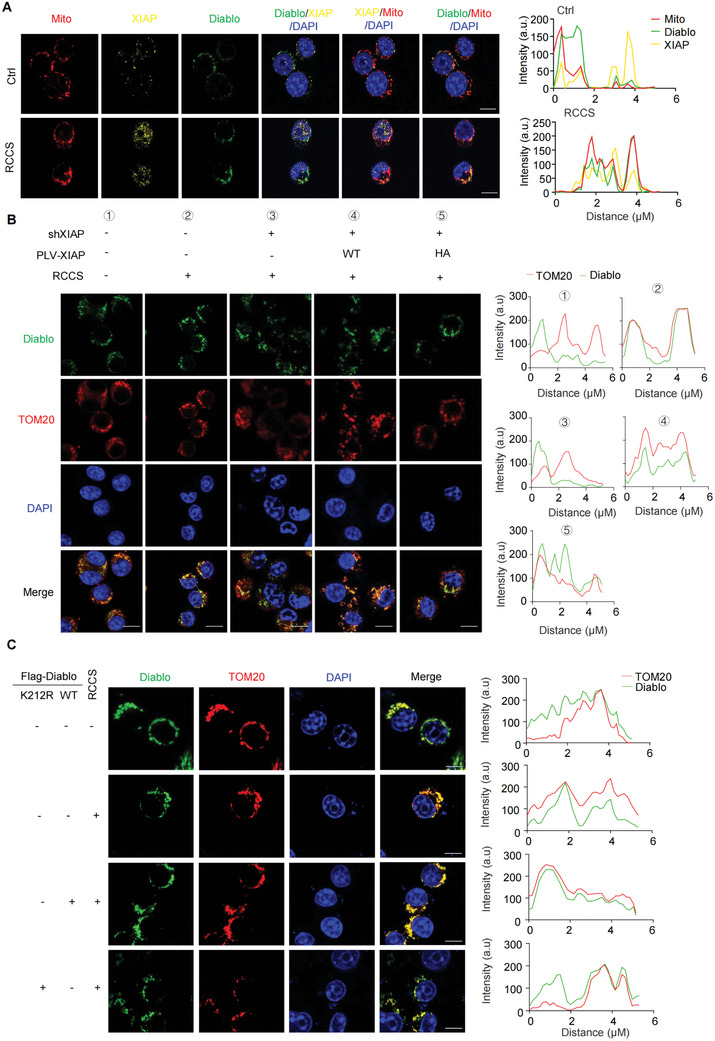
XIAP inhibited Diablo release into cytosol from mitochondrial in response to unloading exposure. A) Immunofluorescence staining of XIAP and Diablo in RAW264.7 cells treated with RCCS. B) Mitochondria were labeled with Mito‐Tracker Red CMXRos and visualized with fluorescent imaging (left). Fluorescence intensity was plotted along the arrows. Quantitative analysis was performed by ImageJ (right). Scale bar, 10 µm. *n* = 3 per group. B) The RAW264.7 cells with XIAP knockdown were stably overexpressed XIAP WT or H466A mutant. Cells were cultured with RCCS for 5 d, followed by immunofluorescence staining of XIAP and TOM20. Scale bar, 10 µm. Quantitative analysis (right) was performed to assess the colocalization of Diablo (green) and mitochondrial (TOM20, red). *n* = 3 per group. C) The RAW264.7 cells with Diablo WT or K212R mutant overexpression were cultured with RCCS for 5 d, followed by immunofluorescence staining of Diablo and TOM20. Fluorescence intensity was quantified analysis was performed by ImageJ (right). Scale bar, 10 µm. *n* = 3 per group.

Next, we went to investigate the impact of XIAP‐mediated K63‐linked ubiquitylation on Diablo in osteoclast lineage cells. Half‐life assays showed that the protein stability of Diablo exhibited no change in the Raw264.7 cells with XIAP overexpression or knockdown compared with the controls (Figure S3K,L, Supporting Information), suggesting that XIAP promoted non‐degenerative ubiquitylation of Diablo at the site of K212 in osteoclast lineage cells. Diablo/SMAC is mainly localized at the inner membrane space of mitochondrial. It is synthesized as a precursor molecule of 239 amino acids; the N‐terminal 55 residues serve as the mitochondria targeting sequence that is removed after import.^[^ ^20^, ^21^ ^]^ Upon apoptotic induction and mitochondrial outer membrane permeabilization (MOMP), Diablo/SMAC, together with cytochrome c (Cyto c) is released into the cytosol where it binds to XIAP, resulting in the release of XIAP‐bound‐caspase 9.^[^ ^18^, ^19^ ^]^ So, we asked if K63‐linked ubiquitylation governed the localization of Diablo within mitochondrial. Immunofluorescence (IF) assay indicated that culture with RCCS, but not RANKL stimulation, enhanced the co‐localization of XIAP and Diablo in mitochondrial (**Figure** **3**A; Figure S4A,B, Supporting Information). XIAP depletion by shRNA significantly promoted Diablo release into cytosol in RCCS‐cultured cells (Figure 3B; Figure S4C, Supporting Information). The reintroduction of XIAP, but not XIAP H466A mutant successfully restored Diablo localization to mitochondrial (Figure 3B; Figure S4C, Supporting Information). Consistently, XIAP knockdown significantly enhanced cell apoptosis (Figure S4D, Supporting Information), and inhibited osteoclasts differentiation with decreased multinucleated TRAP+ cells in RCCS‐cultured cells with RANKL treatment (Figure S4E,F, Supporting Information).

Further, we demonstrated that K212R mutation in Diablo promoted its release into cytosol in RCCS‐cultured cells (Figure 3C). Thus, we revealed unrecognized mechanism by which unloading exposure inhibited intrinsic pathway in osteoclast lineage cells. Briefly, in response to reduced mechanical stress, XIAP facilitated K63‐linkage ubiquitylation on Diablo at the K212 site and hindered Diablo release into cytosol from mitochondrial, thereby suppressing cell apoptosis.

### 2.4 SLC1A5‐Mediated Gln Uptake Is Essential for the Survival of Monocytes DURING Mechanical Unloading

**Figure 4:**
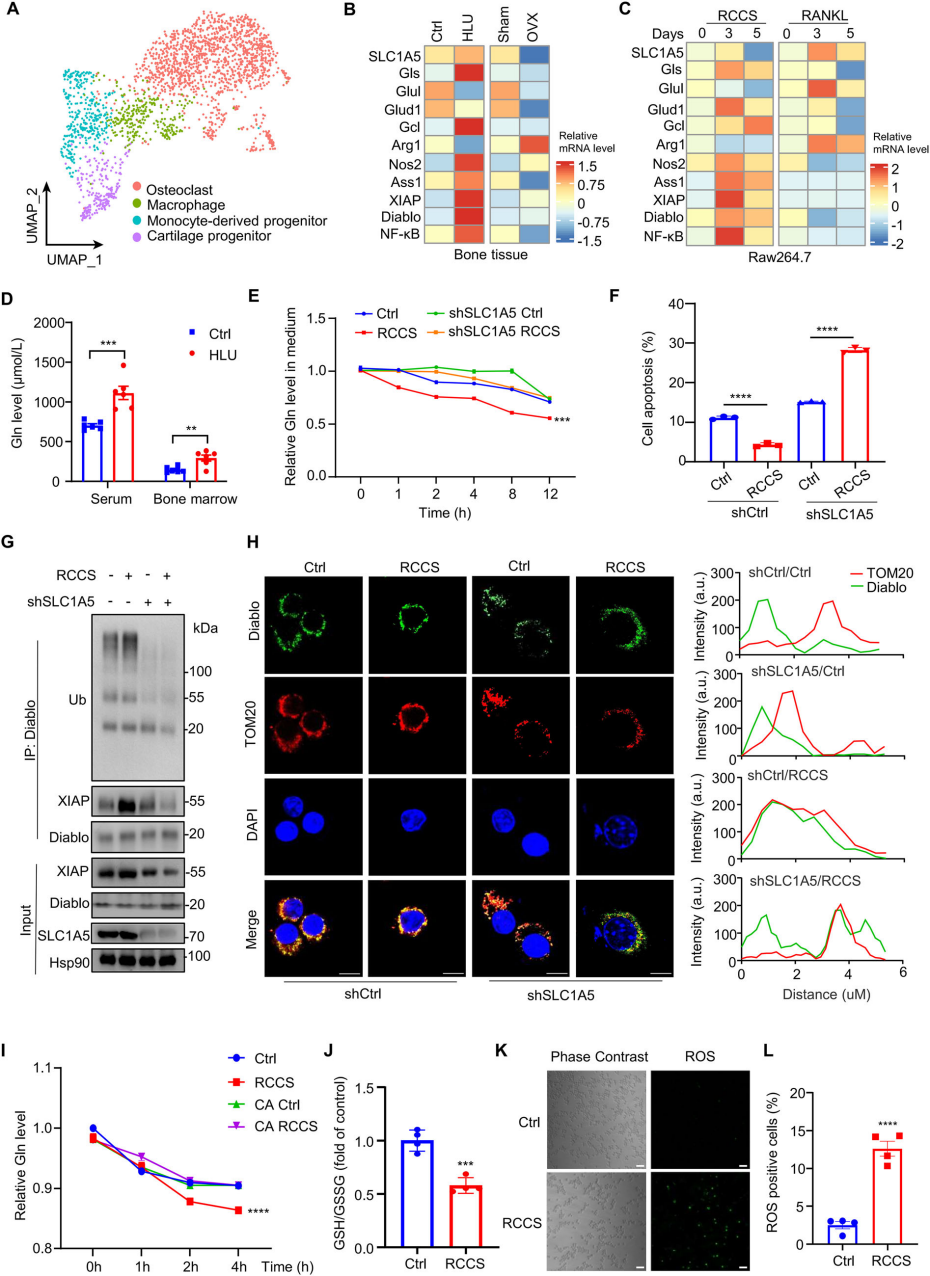
SLC1A5‐mediated Gln uptake is essential for the survival of osteoclast lineage cells during mechanical unloading. A) scRNA‐Seq data were analyzed and subclusters of monocyte were graphically displayed by UMAP plot. *n* = 2 per group. B,C) Q‐PCR was performed in bone tissue from HLU or ovariectomy (OVX) mice and Raw264.7 cells cultured with B) RCCS or C) RANKL. The relative mRNA expression of indicated genes was displayed in the heatmaps. *n* = 4 per group. D) Gln concentration were examined in peripheral blood or bone marrow from HLU and Ctrl mice. *n* = 6 per group. E) SLC1A5 was silenced by shRNA in RAW264.7 cells, followed by RCCS culture for indicated times. Gln uptake assay was conducted to examine the Gln concentration in the cultural supernatant of cells (E, *n* = 6 per group). F) Cell apoptosis was detected by Annexin V staining and flow cytometry (F, *n* = 3 per group). G) SLC1A5 was silenced by shRNA in RAW264.7 cells, followed by RCCS culture for indicated times. The cell lysates were collected for immunoprecipitation assay and immunoblot analysis. H) Immunofluorescence staining of Diablo and TOM20 were conducted,  quantitative analysis of Diablo (green) and mitochondrial (Tom20, red) colocalization (right). Scale bar, 10 µm. *n* = 3 per group. I) The SLC1A5‐silenced RAW264.7 cells were stably transfected with SLC1A5 WT or SLC1A5 C481A mutant, treated with RCCS for indicated times, followed by Gln uptake assay. *n* = 4 per group. J) The ratio of reduced to oxidized glutathione (GSH/GSSG) were assessed in RAW264.7 cells cultured with or without RCCS treatment. *n* = 4 per group. K) Representative images from immunofluorescence staining of reactive oxygen species (ROS) were assessed in RAW264.7 cells cultured with or without RCCS treatment. L) Fluorescence intensity was quantified using Image J. *n* = 4 per group. *P < 0.05, **P<0.01, ***P < 0.001, ****P < 0.0001, n.s., not significant. Data are mean ± SEM. Statistical differences were determined using Student's t‐test.

Subsequently, we went to unveil the underlying mechanism of osteoclasts perceiving mechanical perturbations. We reclustered the monocytes into 4 distinct subpopulations: monocyte‐derived progenitor (MODP) expressing high levels of Cx3cr1, Prtn3, Ly6c2, Lyz2 (but low Spp1 and Ccr2 expression); macrophages expressing Ly6c2, Lyz2, Crip1, Spp1 and S100a4; pre‐osteoclast with high levels of Ctsb, Ccl3, Tnf expression; and a cartilage progenitor cluster expressing Birc5, Ube2c, and Cdkn3 (**Figure** **4**A; Figure S5A,B, Supporting Information). We used the monocle2^[^ ^35^ ^]^ to define differentiation trajectories from the 4 subpopulations by mapping the gene expression changes that accompany monocytes differentiation into osteoclasts. This analysis defined the MODP subset as a starting point and suggested a developmental trajectory from MODP via macrophage toward a pre‐osteoclast population (Figure S5C, Supporting Information). Macrophages split into two branches, the first of which was of pre‐osteoclast lineage while the second branch was cartilage progenitor lineage (Figure S5C, Supporting Information). Cartilage stem/progenitor cells (CSPCs) reside within articular cartilage and are thought to respond to injury and migrate through the disease‐affected tissue zone.^[^ ^36^ ^]^ However, the origin and functions of CSPCs are incompletely understood. Functional enrichment analysis of DEG showed that the terms for amino acid metabolism and glycolysis pathways were enriched in these 3 osteoclast lineage cell groups, MODP, macrophage and pre‐osteoclast (Figure S5D, Supporting Information). The metabolomics results revealed significant alterations in the metabolic intermediates of the Gln pathway within the bone tissue of HLU mice (Figure S5E,F, Supporting Information). Accordingly, significant alterations in the mRNA level of genes relevant to Gln metabolism were observed in osteoclast lineage cells from HLU mice (Figure S5G,H, Supporting Information). The expression of glutaminase (Gls), glutamate dehydrogenase 1 (Glud1) and glutamate cysteine ligase (Gclc), associated with Gln catabolism, were significantly upregulated in bone tissue from HLU mice (Figure 4B), as well as the osteoclast lineage cells with RCCS culture (Figure 4C) compared with the controls. Gls activity was enhanced in RCCS‐cultured Raw264.7 cells (Figure S6A, Supporting Information). Interestingly, Gln concentration was dramatically increased in both peripheral blood and bone marrow from HLU mice compared with the controls (Figure 4D). The level of Gln in the culture supernatant of Raw264.7 cells, but not MC3TCE1 cells, was gradually decreased during RCCS culture, implying the increased Gln influx during RCCS culture (Figure 4E; Figure S6B, Supporting Information).

Na^+^‐dependent transporter SLC1A5 is a Gln transporter and localized in the plasma membrane.^[^ ^37^ ^]^ The blockade of SLC1A5 by shRNA significantly inhibited Gln uptake and downregulated the expression of genes associated with Gln catabolism in Raw264.7 cultured with RCCS (Figure S6C,D, Supporting Information). The inhibitory effect of RCCS culture on cell apoptosis was dramatically reversed in Raw264.7 with SLC1A5 knockdown (Figure 4F; Figure S6E–H, Supporting Information). Consistently, SLC1A5 depletion suppressed XIAP upregulation and the ubiquitin conjugation on Diablo (Figure 4G), thereby promoting Diablo release from mitochondrial to cytosol in monocytes during RCCS culture (Figure 4H; Figure S6I, Supporting Information). To address how SLC1A5 perceive mechanical perturbation, we examined SLC1A5 expression in Raw264.7 cells during mechanical unloading. The SLC1A5 level and membrane localization remain unchanged in Raw264.7 cells cultured with RCCS and BMDM derived from HLU mice (Figure S6J–L, Supporting Information). SLC1A5 had been demonstrated to serve as “redox sensor.”^[^ ^19^, ^24^ ^]^ The Cys residues, particularly Cys467 (C467) in SLC1A5 are essential for sensing redox state of microenvironment and the S–S/SH interconversion of C467 switches it from “OFF” to “ON” state.^[^ ^21^, ^22^, ^38^, ^39^, ^40^ ^]^ To determine if the SLC1A5‐mediated Gln influx upon unloading exposure is dependent on the sensitivity on redox state, we reintroduced SLC1A5 C481A mutant in RAW264.7 cells with SLC1A5 knockdown. C481A mutation effectively inhibited Gln influx triggered by mechanical unloading (Figure 4I), suggesting that SLC1A5 might sense mechanical perturbation in a redox‐dependent manner. To gain insight the redox changes in the osteoclast lineage cells, we examined the metabolic changes in RAW264.7 cells and the culture medium treated with RCCS. In cells, glutathione (GSH) metabolism pathway is significantly enriched (Figure S7A,B, Supporting Information). In the culture medium, alterations in the tyrosine metabolic pathway associated with oxidative stress were observed (Figure S7C, Supporting Information). In addition, the in vitro experiments showed that the ratio of GSH/GSSG was significantly reduced (Figure 4J). Given that GSH serves as the primary cellular antioxidant, fluctuations in its level are linked to intracellular redox dynamics, particularly considering the critical role of reactive oxygen species (ROS) in bone resorption processes.^[^ ^41^, ^42^ ^]^ ROS levels were measured in RAW264.7 cells subjected to RCCS treatment, and significant induction of ROS was observed in the RCCS‐treated group (Figure 4K,L). These data suggest cellular metabolic reprogramming occurs with RCCS which increases ROS and alters aspects of cellular GSH metabolism.

**Figure 5:**
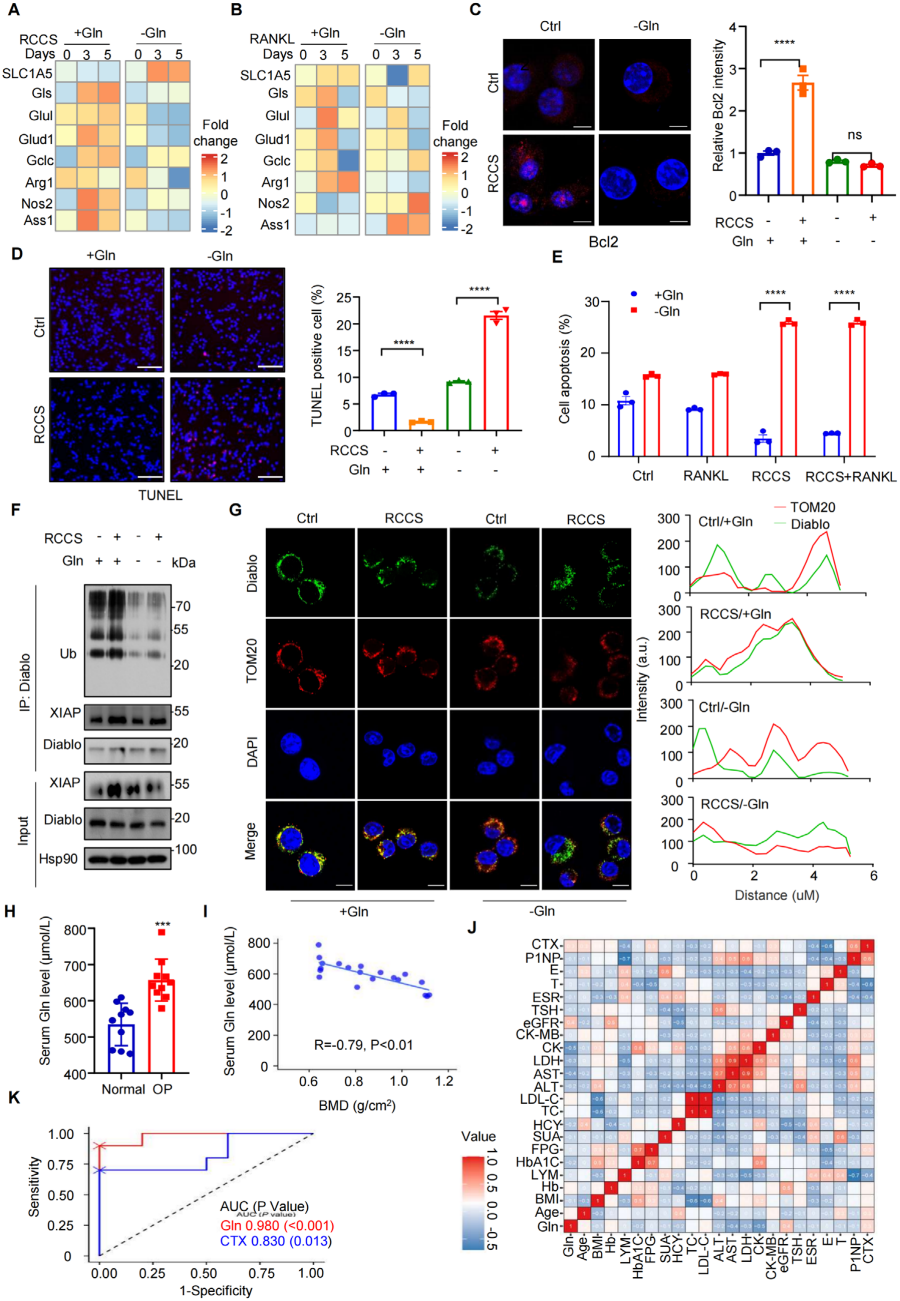
The uptake of glutamine is crucial for the survival of osteoclast lineage cells, and circulating glutamine levels are essential for osteoporosis. A,B) mRNA level of indicated genes was detected by Q‐PCR. The heatmap was used to illustrate relative expression of these genes in RAW264.7 cells cultured with A) RCCS and B) RANKL. *n* = 3 per group. C,D) Representative images from immunofluorescence staining of Bcl‐2 (C, fluorescence intensity was quantified on the right), TUNEL assay (D, fluorescence intensity was quantified on the right) in RCCS‐treated RAW264.7 cells are presented. E) Apoptosis was assessed by Annexin V staining followed by flow cytometric analysis. Scale bars: C) 10 µm, D) 50 µm. *n* = 3 per group. F) The cell lysates were collected from RAW264.7 cells cultured with RCCS for immunoprecipitation assay and immunoblot analysis. *n* = 3 per group. G) The colocalization of Diablo (green) and mitochondria (TOM20, red) in RAW264.7 cells following 0 and 5 d of RCCS treatment in normal medium or Gln‐deprivation DMEM medium, followed by immunofluorescence staining with Diablo and TOM20. Scale bar, 10 µm. Quantitative analysis was performed by ImageJ (right). *n* = 3 per group. H) Serum levels of Gln in participants in the normal and osteoporosis (OP) groups (*n* = 10, respectively). I) Association between serum level of Gln and bone mineral density (BMD) of the femoral neck. *R* and *P* value from linear regression analyses. *n* = 10 per group. J) ROC curve of Gln (red) and CTX (blue) for OP. Gln: the mean area under the ROC curve was 0.980. CTX: the mean area under the ROC curve was 0.830. AUC, area under the curve; ROC, receiver operating characteristic. *n* = 10 per group. K) Correlation matrix between Gln and age, body mass index (BMI) and other clinical features. Red blocks show positive correlations while blue blocks show negative correlations. The number in the blocks indicates the correlation score. *n* = 10 per group. *P < 0.05, **P < 0.01, ***P < 0.001, ****P < 0.0001, n.s., not significant. Data are mean ± SEM. Statistical differences were determined using Student's t‐test.

Gln has been documented to be the most abundant non‐essential amino acid in the plasma and serves as a carbon and nitrogen donor for the production of biomolecules.^[^ ^20^ ^]^ It can be synthesized endogenously but becomes “conditionally essential” in physiological or pathological conditions of high proliferation rate.^[^ ^20^ ^]^ To validate the indispensability of Gln uptake on the survival of osteoclast lineage cells under unloading exposure, we cultured Raw264.7 cells in the medium without Gln and found that Gln deprivation inhibited the upregulation of the mRNA level of Gln catabolism‐associated genes induced by RCCS culture (**Figure** **5**A,B). Notably, under Gln deprivation, RCCS culture induced a substantial increase of cell apoptosis (Figure 5C–E). The activities of Caspase 3 and 9 were significantly increased in RCCS treatment cells cultured with Gln‐deprivation medium (Figure S8A,B, Supporting Information). However, during only RANKL‐induced osteoclastogenesis, cell apoptosis remains unchanged in cells with Gln deprivation (Figure S8C, Supporting Information). Consistently, Gln‐deprivation impaired osteoclast differentiation and inhibited bone resorption (Figure S8D,E, Supporting Information). Diablo ubiquitylation and accumulation in mitochondrial induced by RCCS culture were significantly attenuated in cells cultured with Gln‐deprivation medium (Figure 5F,G; Figure S8F,G, Supporting Information). In addition, we observed that the formation of mitochondrial permeability transition pore (MPTP) was suppressed in the absence of Gln, suggesting that Gln‐deprivation enhanced mitochondrial permeability in Raw264.7 cells with RCCS treatment (Figure S8H, Supporting Information). This contributed to Diablo release into cytoplasm in absence of Gln. Therefore, we proposed that SLC1A5‐mediated Gln uptake is essential for the survival of monocytes during mechanical unloading.

### 2.5 Targeting the Gln/SLC1A5 Axis Effectively Preserved Bone Mass under Mechanical Unloading

To extend the clinical relevance of Gln level to osteoporosis, we randomly recruited 20 sedentary males over the age of 65 years, comprising 10 individuals with normal bone mineral density (BMD) and 10 individuals diagnosed with osteoporosis (OP). The clinical characteristics of eligible participants are summarized in Table S4, Supporting Information, and no significance was found in the age, BMI, common chronic diseases and lifestyles (e.g., diets, exercise, smoking, drinking, tea, milk, and eggs) between OP and normal groups. The serum Gln level in OP group was higher than normal group (656.87±58.08 vs 534.57±58.50 µmol L^−1^, *P* < 0.001) (Figure 5H). Gln levels tended to decrease with the increase of femoral neck BMD (*r* = 0.79, *P* < 0.001) (Figure 5I). Correlations between Gln levels and other clinical features were also estimated and found that Gln levels were positively correlated with cross linked C‐telopeptide of type I collagen (CTX) levels and negatively correlated with P1NP levels (Figure 5J). We further validated the predictive power of Gln and CTX levels for OP, and significant difference in the area under the curve (AUC) was observed (0.980 vs 0.780) (Figure 5K), indicating that Gln levels in circulation might serve as a potential biomarker for predicting the risk of osteoporosis.

**Figure 6:**
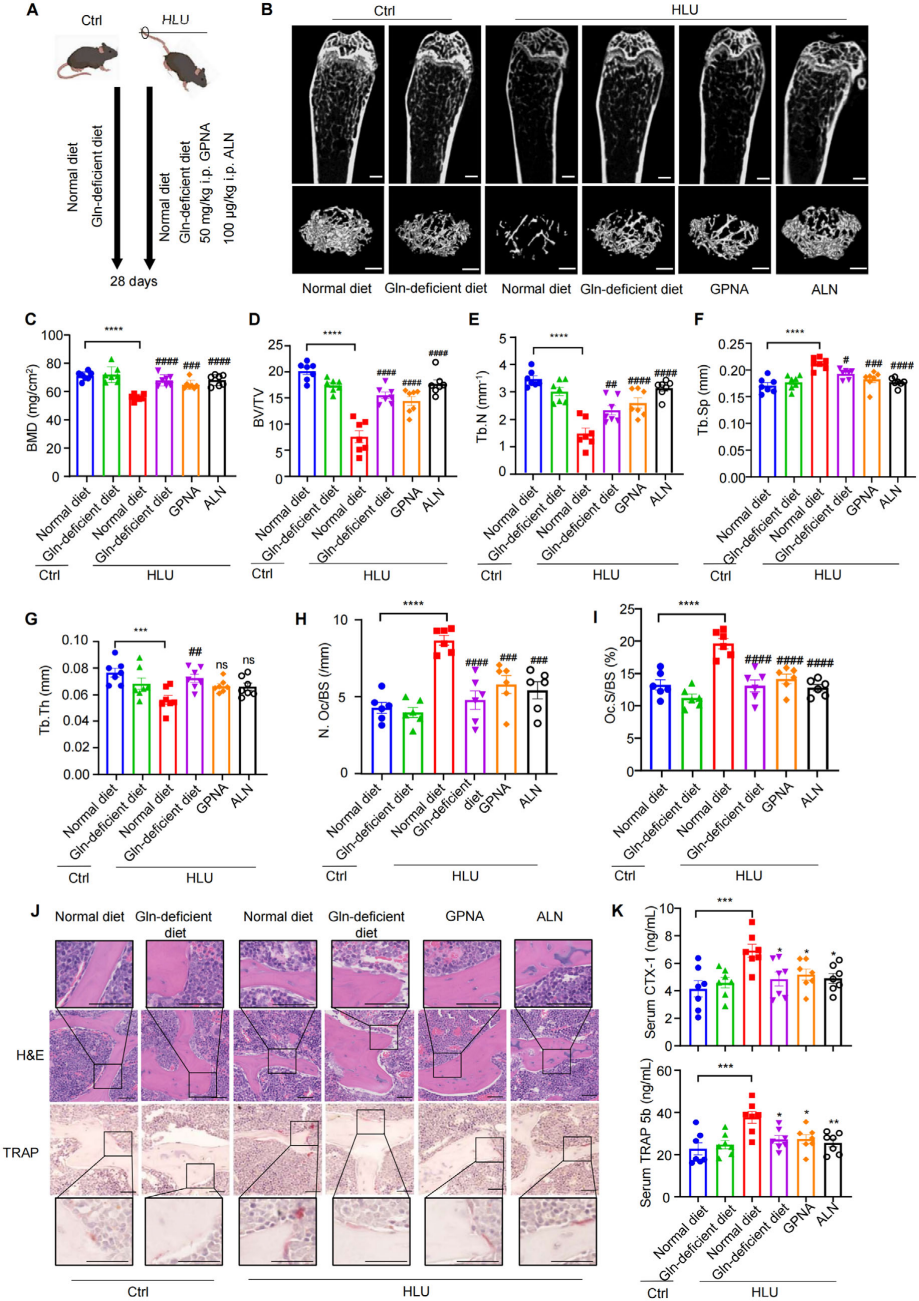
Blocking Gln uptake effectively preserved bone mass under mechanical unloading. A) Schematic diagram of HLU induced osteoporosis in mice with indicated treatments. *n* = 7 per group. B) Representative micro‐CT images of whole femoral (top) and trabecular (bottom) bones. *n* = 7 per group. Scale bars, 0.5 mm. C–G) Histomorphometric analysis of trabecular bones, including C) bone mass density (BMD), D) bone volume per tissue volume (BV/TV), E) trabecular number (Tb.N), F) trabecular spacing (Tb.Sp) and G) trabecular thickness (Tb.Th). *n* = 7 per group. H,I) Histological analysis the number of H) osteoclasts (N.Oc/BS) and I) osteoclasts surface (Oc.S/BS) per unit of trabecular bone surface of femurs from mice. *n* = 6 per group. J) Representative images of staining with hematoxylin–eosin (H&E) and Tartrateresistant acid phosphatase (TRAP) of femurs from indicated mice. High‐magnification images of the same area are shown in the top and bottom. Scale Bar: 50 µm. *n* = 6 per group. K) Mouse serum CTX‐1 and TRAP 5b, which reflect bone resorption, were detected by ELISA. *n* = 7 per group. *P < 0.05, **P < 0.01, ***P < 0.001, ****P < 0.0001, n.s., not significant. Data are mean ± SEM. #*P* < 0.05, ##*P* < 0.01, ###*P* < 0.001, ####*P* < 0.0001 versus HLU with normal diet. n.s., not significant. Statistical differences were determined using ANOVA.

**Figure 7:**
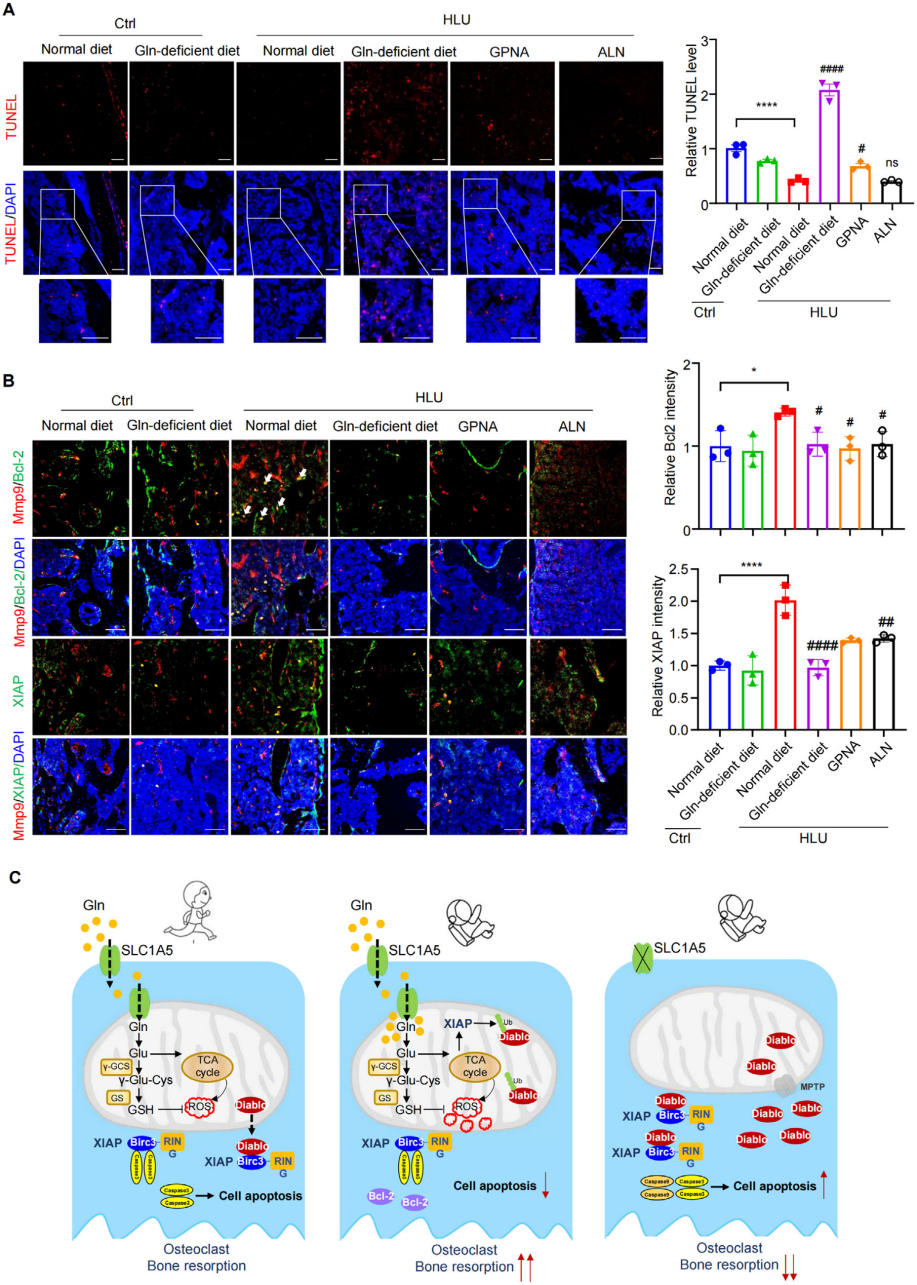
Blocking Gln influx effectively inhibited the intrinsic apoptotic pathway of osteoclast lineage cells during mechanical unloading. A) Representative images displayed apoptosis of femurs from different treatment mice were detected by TUNEL staining. Inset shows TUNEL staining at a higher magnification. Quantitative analysis was performed (right). Scale, 50 µm. *n* = 3 per group. B) Immunofluorescence staining of Bcl‐2, Mmp9 and XIAP in femurs from mice. The nuclei were stained with DAPI. Scale bar, 50 µm. Quantitative analysis of Bcl‐2 (top, green) and XIAP (bottom, green) relative intensity were displayed in histograms (right). *n* = 3 per group. C) The proposed working model of Gln catabolism‐driven apoptotic suppression in osteoclasts upon unloading exposure. *P < 0.05, **P < 0.01, ***P < 0.001, ****P < 0.0001, n.s., not significant. *n* = 3 per group. Data are mean ± SEM. #*P* < 0.05, ##*P*<0.01, ###*P* < 0.001, ####*P* < 0.0001 versus HLU with normal diet. n.s., not significant. Statistical differences were determined using ANOVA.

Then we sought to explore whether blocking Gln influx could attenuate unloading‐induced bone loss. Gln uptake through the transporter SLC1A5 had been found stimulated in many cancer models.^[^ ^43^ ^]^ This metabolic feature has prompted various attempts to inhibit Gln entry as a device to hinder cancer cell proliferation. L‐γ‐Glutamyl‐p‐nitroanilide (GPNA) was proposed as an SLC1A5 inhibitor and has been subsequently widely used to this purpose.^[^ ^44^, ^45^, ^46^ ^]^ HLU mice were treated with GPNA or vehicle for 4 weeks (**Figure** **6**A). The quantitative computed tomography (µ‐QCT) analysis showed that trabecular bone mass was significantly increased in mice treated with GPNA compared to vehicle (Figure 6B), as confirmed by increased bone mineral density (BMD, Figure 6C), trabecular bone volume (BV/TV, Figure 6D), trabecular number (Tb. N, Figure 6E) and trabecular thickness (Tb.Th, Figure 6G). And decreased trabecular spacing (Tb. Sp, Figure 6F). In addition, we employed Gln‐deficient diet to validate the role of exogenous Gln in unloading‐induce bone resorption (Figure 6A). Compared with normal diet, Gln‐deficient diet remarkably increased trabecular bone mass of HLU mice (Figure 6B–G), while the bone mass of control mice fed with a Gln‐deficient diet remained unaffected (Figure 6B–G). H&E and TRAP staining exhibited a significant decrease in the number of osteoclasts from mice treated with GPNA as well as fed with Gln‐deficient diet (Figure 6H–J; Figure S9A,B, Supporting Information). In addition, serum levels of the bone resorption markers CTX‐1 and TRAP 5b were measured. Following HLU modeling, both markers were significantly decreased in the GPNA group and in mice fed a Gln‐deficient diet compared to the normal diet group (Figure 6K). Accordingly, we observed a significant inhibition of the intrinsic apoptotic pathway, as indicated by TUNEL, Bcl‐2, and XIAP staining in the bone tissues from all treatment groups mentioned above (**Figure** **7**A,B; Figure S9C, Supporting Information). Treatment with GPNA and Gln‐deficient diet inhibited Diablo ubiquitylation in bone tissues from HLU compared with the vehicle control (Figure S9D, Supporting Information). Interestingly, we observed elevated levels of Gln in both serum and bone marrow samples from HLU mice compared to the control group. However, these elevated Gln levels were restored to normal when mice were treated with GPNA or fed with a Gln‐deficient diet (Figure S9E,F, Supporting Information). The mRNA expression of Gls, Glud1, and Gclc in bone tissue were decreased in the bone tissues from HLU mice treated with GPNA or Gln‐deficient diet (Figure S9G–I, Supporting Information), indicating that Gln catabolism was suppressed in the treatment group of mice. Thus, we provided potential therapeutic drugs and strategies for osteoporosis resulted from reduced mechanical force.

## 3 Discussion

In this study, through integrating multiple omics data of bone tissues from HLU and control mice, we revealed that BMDMs were characterized by suppressed intrinsic apoptotic pathway and augmented Gln catabolism in the absence of mechanical force. We discovered that the utilization of Gln is essential for the survival of monocytes under mechanical unloading. Additionally, we unveiled that ubiquitin E3 ligase XIAP facilitated ubiquitylation of Diablo/SMAC and inhibited its release into the cytosol upon mechanical unloading, ultimately leading to the suppression of osteoclast apoptosis in a Gln‐dependent manner. Importantly, we demonstrated that targeting Gln transport through SLC1A5 inhibitor or Gln‐deficient diet, effectively preserved bone mass in HLU mice, implicating attractive approaches for combatting bone loss induced by weightlessness or disuse (Figure 7C).

Ubiquitin is covalently attached to a lysine residue on a protein substrate in a reaction involving ubiquitin‐activating enzymes (E1), ubiquitin‐conjugating enzymes (E2) and ubiquitin ligases (E3).^[^ ^47^, ^48^, ^49^ ^]^ A global increase in poly ubiquitin conjugation has been observed during the cellular response to many types of stress.^[^ ^50^, ^51^, ^52^ ^]^ Here, we generated integrative analysis framework for proteome and ubiquitinome, and provided unbiased insights into the protein homeostasis in stress response by connecting differentially ubiquitinated proteins with specific biological pathways. We predicted degradative ubiquitylation of 27 sites in 20 proteins and non‐degradative modification of 864 sites in 500 proteins. We systematically mapped an ubiquitylation‐relevant PPI network between E3/DUB and substrates and predicted total of 277 E3 ligase‐substrate interactions (ESIs) and 5 deubiquitylase‐substrate interactions (DSIs). The present study represents the initial attempt to depict the landscape of ubiquitin modification in bone tissue in response to mechanical stress.

Additionally, we revealed that mechanical unloading significantly promoted the survival in monocytes by inhibiting the intrinsic apoptotic pathway. We unveiled previously unrecognized mechanism by which E3 ligase XIAP governed the subcellular localization of Diablo in mitochondrial through catalyzing K63‐linked ubiquitylation at the K212 site of Diablo, ultimately inhibiting cell apoptosis. XIAP has been established to be negatively regulated by Diablo which binds to the caspase pocket in BIR3 domain of XIAP, resulting in the release of XIAP‐bound‐caspases and cell apoptosis.^[^ ^15^, ^16^, ^17^, ^18^ ^]^ This finding provided a novel insight into the mode of action of the XIAP/Diablo complex in mitochondrial of osteoclast lineage cells.

Gln is the most abundant non‐essential amino acid in the plasma and serves as a carbon and nitrogen donor to produce biomolecules.^[^ ^20^ ^]^ We established the relationship between Gln utilization and the perception to mechanical perturbations for the first time. Gln transporter SLC1A5 might serve as a sensor for mechanical unloading in osteoclast lineage cells and transmits mechanical perturbation into biological effect through regulating Gln influx. Clinically, we reported that the serum Gln levels in OP individuals were higher than normal group, and might serve as a potential biomarker for predicting the risk of osteoporosis. Further, we provided attractive approaches for counteracting bone loss induced by weightlessness or disuse. Targeting the Gln uptake through SLC1A5 inhibitor GPNA, as well as Gln‐deficient diet, effectively preserved bone mass in HLU mice. Particularly, Gln‐deficient diet appears to be a safer and more effective strategy for combatting bone resorption. Our findings will develop therapeutic strategies for disuse osteoporosis in the setting of prolonged bed rest or exposure to long‐term microgravity environment in space.

## 4 Conclusion

We depicted the landscape of ubiquitin modification in bone tissue in response to mechanical stress and reported a previously unrecognized mechanism underlying apoptotic suppression in monocytes during mechanical unloading. We discovered that ubiquitin E3 ligase XIAP facilitated the K63‐linkage ubiquitylation and mitochondrial localization of Diablo, leading to the suppression of monocytes apoptosis. Additionally, we revealed that SLC1A5‐mediated Gln uptake was essential for the survival of monocytes during mechanical unloading. The blockade of Gln uptake impedes Diablo ubiquitylation and promotes the cytosolic release of Diablo during mechanical unloading. Targeting Gln transport through SLC1A5 inhibitor or Gln‐deficient diet, effectively preserved bone mass in HLU mice, implicating attractive approaches for combatting bone loss induced by weightlessness or disuse.

## 5 Experimental Section

### Mouse Models

All experimental procedures were approved by the Institutional Animal Care and Use Committee of the Beijing Institute of Lifeomics (IACUC‐DWZX‐2022‐630).

Eight‐week‐old wild‐type C57BL/6J male (23±1.5 g) or female (20±1.5 g) were obtained from GemPharmatech Co., Ltd. (XM000975). They were individually tail‐suspended using an overhead pulley system in customized cages to create a hindlimb unloading condition. The unloading height was adjusted to maintain the mice's bodies at approximately 30° from the horizontal plane. The mice were subjected to hindlimb unloading (HLU) for 28 d, and control (Ctrl) group was housed under the same conditions without tail suspension. The male mice in the glutamine‐deficient diet group were provided with a glutamine‐deficient diet of food (Biopike, M11708). The male mice in the SLC1A5 inhibitor group were intraperitoneally injected with 50 mg kg^−1^ L‐γ‐Glutamyl‐p‐nitroanilide (GPNA, Psaitong) diluted in 10% DMSO, once every two days. Male mice in the alendronate (ALN, Psaitong) treatment group were injected intraperitoneally with 100 µg kg^−1^ ALN diluted in phosphate‐buffered saline (PBS), twice a week. The control group received an equal volume of vehicle. After four weeks of treatment, bilateral femurs and tibias were collected for subsequent experiments.

Eight‐week‐old wild‐type C57BL/6J female (20±1.5 g) were obtained from GemPharmatech Co., Ltd. (XM000975). Before surgery, all mice were fasted for 12 h. 5 min prior to the procedure, isoflurane inhalation pre‐anesthesia was administered, followed by continuous inhalation anesthesia throughout the entire surgical process. Abdominal hair was removed, the area disinfected with iodophor. A lateral abdominal incision exposed the abdominal cavity, which was dissected layer by layer. The ovary, enclosed in adipose tissue at the uterine horn, and Fallopian tube were visualized. The fallopian tube was ligated with sutures, and the ovary excised using forceps. Following surgery, the peritoneum and skin were sutured sequentially, the incision re‐disinfected, and animals placed in a cage to recover. Three days postoperatively, animals received a 20 000 unit IM injection of penicillin G sodium to prevent infection and were monitored for complications. The mice were sacrificed 4 weeks after surgery, uterine atrophy was first confirmed, followed by bone collection for µCT and histological analyses.

### Cell Lines

RAW264.7 cells (ATCC, TIB‐71) were cultured in Dulbecco's modified Eagle's medium (DMEM, Gibco, C11995500BT‐1) or glutamine‐free DMEM (Pricella, PM150213) supplemented with 10% fetal bovine serum (FBS, Gemini, 900‐108), 1% (v/v) penicillin/streptomycin (CellWorld, C0160‐611). HEK293T (ATCC, CRL‐3216) were cultured in DMEM (Gibco, C11995500BT‐1) supplemented with 10% fetal bovine serum (FBS, Cellmax, SA301.02.V). MC3T3‐E1 cells (ATCC, CRL‐2594) were maintained in α‐MEM (Gibco, C12571500BT) with 10% FBS (Gemini, 900‐ 108). The cell lines were routinely tested negative for mycoplasma contamination.

### BMDMs Isolation and Culture

Bone marrow cells were isolated from tibiae and femurs of wild‐type C57BL/6J male mice. 2 mL erythrocyte lysate was added to the obtained cells. The cells were then flushed and cultured on dishes in DMEM containing 10% fetal bovine serum and 10 ng mL^−1^ M‐CSF (Novoprotein, CB34) for 3 d. After 3 d, the culture medium was changed, and new M‐CSF (20 ng mL^−1^) was added. The cells were cultured until they fully attached to the dish, resulting in bone marrow‐derived macrophages (BMDMs).

### BMSCs Isolation and Culture

Bone marrow cells were isolated from tibiae and femurs of eight‐week‐old wild‐type C57BL/6J male mice. 2 mL erythrocyte lysate was added to the obtained cells. The cells were then flushed and cultured on dishes in α‐MEM containing 10% fetal bovine serum for 3 d. After 3 d, the culture medium was changed, and the cells were cultured until they were fully attached to the dish, resulting in bone marrow mesenchymal stem cells (BMSCs).

### 3D Culture in the RCCS Bioreactor

To create a microgravity culture environment on the ground, we use the Rotary Cell Culture System bioreactor (RCCS, Synthecon). It consists of a disposable vessel with an oxygenator membrane that allows gas exchange. A 5 mL RAW264.7 or MC3T3‐E1 suspension (1×10^6^ cells mL^−1^) and 5 pieces of microcarrier (Cytoniche, W01‐200) were injected into 50 mL disposable vessels, followed by the removal of bubbles using sterile syringes. The vessels were attached to the rotator base set in a 37 °C, 5% CO2 incubator and rotated at 16 to 24 rpm for and exposure times 3 d, followed by culturing RAW264.7cells for 5 d with 30 ng mL^−1^ M‐CSF (Novoprotein, CB34) and 50 ng mL^−1^ RANKL (R&D, 462‐TEC‐010) in T12.5 cell culture flasks. Independent control cells were cultured in cell culture flasks with 30 ng mL^−1^ M‐CSF and 50 ng mL^−1^ RANKL simultaneously. After the microgravity treatment, the cells were acquired by lysing the microcarriers using digest lysate (Cytoniche, R001‐500). For ‐Gln treatment, the medium was replaced with glutamine deprivation DMEM.

### RANKL‐Induced Osteoclastogenesis

For osteoclastogenesis assays, RAW264.7 cells were added to T12.5 cell culture flasks at a seeding density of 6×10^5^ cells per well in triplicate. Cells were incubated with 30 ng mL^−1^ M‐CSF and 50 ng mL^−1^ RANKL for 5 d in DMEM supplemented with 10% FBS. The medium was changed every 2 d. After treatment, qRT‐PCR, cell apoptosis, TRAP staining, western blotting and other experiments were performed.

### Pit Formation Assay

To assess bone resorption, bone slices were labeled, placed label‐side down in 96‐well plates. On the experimental day, cells (10 000 cells per well) treated with RCCS were seeded in normal or glutamine‐free DMEM containing 30 ng mL^−1^ M‐CSF and 50 ng mL^−1^ RANKL for 6 days, with media changed every other day. Slices were fixed in 2.5% glutaraldehyde (in PBS) for 30 min at room temperature, then adherent cells removed by sonication in distilled water (50 Hz, 5 min). Slices were air‐dried on filter paper, stained with 1% toluidine blue (in 1% sodium borate) for 20 min at room temperature, and pit areas quantified in 3 random fields via ImageJ.

### Cell Mitochondrial Separation

Collect cells of different treatments and wash them with PBS. Add the mitochondrial separation reagent (Beyotime, C3601) according to the manufacturer's instructions. Transfer the cell suspension to the homogenizer for homogenization. Subsequently, centrifuge the homogenate at 600 × *g* for 10 min at 4 °C to pellet unbroken cells and nuclei. Transfer the supernatant to a new centrifuge tube and centrifuge at 11 000 × *g* for 10 min at 4 °C to pellet the mitochondria. The resulting supernatant represents the cytoplasmic fraction, while the pellet contains the isolated mitochondrial fraction, which is subjected to boiling for western blotting.

### ROS Detection

Collect cells of different treatments in 48‐well plate. For determination of ROS production, cells were treated with 10 µm 2′,7′‐dichlorodihydrofluorescein (DCFH‐DA) (Beyotime, S0033S) as manufacturer's protocols. The fluorescence intensity of 2′,7′‐dichlorofluorescein (DCF) was observed directly with a laser confocal microscope (Zeiss, LSM880).

### Mitochondrial Permeability Transition Pore (MPTP) Detection

MPTP was detected by an MPTP Assay Kit (Beyotime, C2009S). Cells from different treatment groups were seeded into confocal dishes, washed with PBS, and incubated with calcein AM and Co^2^⁺ quencher at 37 °C for 30 min. The dye solution was then replaced with fresh culture medium, and slides were further incubated at 37 °C for 30 min in the dark before fluorescence microscopy analysis (Zeiss, LSM880).

### Caspase9 Activity Assay

Caspase 9 activities were assayed using a kit (Beyotime, C1157). Briefly, cells from different treatment groups were collected and rinsed with cold PBS, then lysed by lysis buffer for 15 min on ice. Cell lysates were centrifuged at 20 000×*g* for 15 min at 4 °C. The reaction system was set up according to the manufacturer's instructions, followed by the addition of the cell lysates and incubation at 37 °C for 120 min. A405 was measured once a noticeable color change occurred.

### Glutathione Measurement

Glutathione (GSH) and glutathione, oxidized (GSSG) levels were measured using a GSH and GSSG Assay Kit (Beyotime, S0053). The reaction system was set up according to the manufacturer's instructions, followed by the addition of the cell lysates and incubation at 25 °C for 60 min. A412 was measured once a noticeable color change occurred.

### Glutamine Assay

The Glutamine/Glutamate‐Glo Assay (Promega, J8021) was used to measure alterations in glutamine and glutamate concentrations in mammalian cell culture medium. Following treatment of RAW264.7 or MC3T3‐E1 cells at consistent cell density, the medium from each treatment was collected at the same time point. Medium from the various treated samples was diluted 70‐fold in PBS, and subsequent experiments were conducted according to the manufacturer's instructions.

### Antibodies, Plasmid Construction and Primers

All antibodies and plasmid constructions are described in Tables S5 and S6, Supporting Information, respectively. The quantitative real‐time PCR (qRT‐PCR) primers are listed in Table S7, Supporting Information. The short hairpin RNA sequences are provided in Table S8, Supporting Information.

### RNA Interference

Use online tools like BLOCK‐iT RNAi Designer or siRNA Wizard to design specific shRNA sequences for the target gene. After synthesizing and cloning the designed shRNA sequence into an appropriate vector, sequencing is necessary to ensure the sequence's correctness. Select stable clones expressing shRNA through antibiotic (puromycin) screening. To validate the downregulation of the target gene expression, employ qRT‐PCR and Western blotting to detect changes in mRNA and protein levels, respectively. The cells with knocked‐down expression were subsequently infected with lentivirus to introduce the overexpressed molecules. Stable expression was selected using Hygromycin, and the presence of the expression was confirmed through qPCR and immunoblotting.

### Cell Transfection and Immunoprecipitation

HEK293T cells (ATCC, CRL‐3216) were transfected with the respective expression plasmids utilizing LipoPlus (Genestar, C101‐01). After 48 h, cell lysis was performed using HEPES lysis buffer (20 mm HEPES (pH 7.2), 50 mm NaCl, 0.5% Triton X‐100, 1 mm NaF, and 1 mm dithiothreitol), supplemented with protease inhibitors (MCE, Lot#278910). The lysate was subsequently centrifuged at 12 000 × *g* for 10 min at 4 °C, and the resulting supernatant was incubated overnight at 4 °C with the respective primary antibody and protein A/G agarose beads (Santa Cruz, #h821). The beads were washed three times with HEPES buffer and subjected to boiling for western blotting.

For ubiquitination studies, cells or tissues were exposed to 20 µm of the proteasome inhibitor MG132 (Sigma‐Aldrich, M8699) for 8 h prior to sample collection. Subsequently, cells or tissues were lysed in RIPA lysis buffer (50 mm Tris (pH 7.5), 150 mm NaCl, 1% NP‐40, 10 mm NaF, and 1 mm Na3VO4), supplemented with protease inhibitor (MCE, Lot#278910). The immunocomplexes were washed multiple times with lysis buffer, resolved by SDS‐PAGE, and immunoblotted with the specified antibodies.

### Protein Half‐Life Assay

Cells were subjected to treatment with cycloheximide (MedChemExpress, HY‐12320) for the specified durations, followed by lysis and subsequent western blot analysis.

### Quantitative Real‐Time PCR (qRT‐PCR)

Total RNA from cells or bone tissues were isolated using Trizol reagent (Tsingke, TSP401). RNA was reverse transcribed to cDNA with the Prime Script RT Reagent Kit (TOYOBO, FSQ201). Gene expression was measured by quantitative PCR (Roche, Basel, Switzerland) using the SYBR‐green PCR Kit (Toyobo, QPK‐201T). The fold changes were calculated using 2−ΔΔCt method.

### Micro‐CT Analysis

Femurs were dissected free of soft tissue, fixed in 10% neutral buffered formalin for 24 h, and then scanned and analyzed with high‐resolution µCT (Quantum GX2, Perkin Elme MicroCT). The scanner operated at a voltage of 50 kV, a current of 100 µA and a resolution of 9 µm per pixel. Bone volume and trabecular bone parameters were calculated from the images of the 0.5 mm thick distal femoral area starting from 0.5 mm below the lowest point of growth plate. 3D reconstructions were generated with software. Bone volume fraction (BV/TV, %), trabecular number (Tb.N, 1 per mm), trabecular thickness (Tb.Th, mm), trabecular separation (Tb.Sp, mm) were assessed using Biomedical Imaging Resource (Rochester, MN) analysis software. Bone mineral density (BMD) was measured using a dual‐energy X‐ray absorptiometry device (Discovery W, Hologic, Waltham, MA, USA).

### Histology and Histomorphometric Analysis

Femurs were harvested from mice after euthanasia on day 28, fixed in 10% formalin for 24 h, decalcified in 10% EDTA for 14 d, and embedded in paraffin for sectioning. Paraffin tissue was sectioned continuously at 5 µm and stained with hematoxylin and eosin (H&E, Solarbio, G1120), and tartrate‐resistant acid phosphatase (TRAP, Wako, 294–67001) according to the manufacturer's instructions. Bone static histomorphometric analyses for the number of osteoblasts per unit of bone surface (N.Ob/BS) and osteoclasts surface per unit of bone surface (Oc.S/BS) were analyzed with the Bioquant Osteo software (Bioquant Image Analysis Corp, USA).

### Enzyme‐Linked Immunosorbent Assay (ELISA)

Quantitative measurements of serum C‐terminal telopeptide of type I collagen (CTX‐1) (CLOUD‐CLONE CORP, UCEA665Mu) and Tartrate‐resistant acid phosphatase 5b (TRACP 5b) (JONLNBIO, JL47637) were performed according to the manufacturer's instructions.

### Immunofluorescence

Femurs were dissected and fixed in 10% formalin for 24 h, applied for frozen embedding and sectioning. Frozen sections of 8 µm thickness were prepared. Sections were fixed with 10% formalin for 10 min, followed by permeabilization in 0.2% Triton X‐100. Sections were blocked with 3% BSA in PBS for 1 h at 37 °C. Subsequently, they were incubated with the indicated primary antibodies overnight at 4 °C. Afterwards, sections were incubated with Alexa Fluor 488/594/647 IgG (Invitrogen) antibodies and 4′‐6‐diamidino‐2‐phenylindole (DAPI, Beyotime) for 1 h at room temperature. Cell samples were fixed in Mito‐Tracker Red CMXRos (Beyotime) and 10% formalin, then stained with indicated antibodies, followed by Alexa Fluor 488/594/647 IgG (Invitrogen). Sections and cells were examined using a confocal microscope (Zeiss, LSM880), and images were captured with image manager software (ZEN3.4).

### Flow Cytometry

Flow cytometry was employed to evaluate apoptosis of the treated cells. Different treated cells were collected and added 100 µL binding buffer. Cells were stained with Annexin V‐Phycoerythrin (PE) /7‐Aminoactinomycin D (7‐AAD) assay kit or Annexin V‐FITC/PI Apoptosis Detection Kit, respectively (Vazyme, China) for 10 min at room temperature. Cells were then added 300 µL binding buffer and analyzed using a flow cytometer (BD FACSVerse). FlowJo version v10.6.2 (Ashland, Oregon) software was used to analyze the data.

### TUNEL

The TUNEL technique was utilized to investigate apoptosis in situ. Fluorometric TUNEL System (Vazyme, China) was used to perform the TUNEL assay on frozen sections of bone tissue and cells. Following fixation with 10% formalin, the sections and cells were processed according to the manufacturer's instructions, including washing, dilution, digestion, and labeling. Sections and cells were examined using a microscope (Zeiss, LSM880), and images were captured with image manager software (ZEN3.4).

### In Vitro Ubiquitination Assay

GST‐Diablo, GST‐XIAP, GST‐XIAP H466A and Ub were expressed in Escherichia coli BL21 (DE3). An in vitro ubiquitination assay was followed by mixing with 0.5 µg of GST‐Diablo and 0.5 µg of GST‐XIAP, E1, E2, and Ub in final volume 50 µL reaction buffer (50 mm Hepes pH 8.0, 100 mm NaCl, 10 mm Mg^2+^ ATP, 0.5 mm DTT) The samples were then incubated at 37 °C for 4 h. The beads were washed three times with lysis buffer, and the reactions were terminated with SDS‐PAGE loading buffer before undergoing immunoblotting (IB) analysis.

### Protein Extraction

Eight‐week‐old wild‐type C57BL/6J male mice subjected to hindlimb unloading (HLU) for 28 d (*n* = 4), and Ctrl group was also caged under the same conditions except for tail suspension (*n* = 4). Femurs were harvested from mice after euthanasia on day 28. The samples were ground with liquid nitrogen into cell powder and then transferred to 5 mL centrifuge tubes. Four volumes of SDC buffer (1% SDC, 10 mm TCEP, 40 mm CAA, 1% protease inhibitor cocktail, 1% phosphatase inhibitor for phosphorylation) were added to the tissue, followed by sonication on ice using a high‐intensity ultrasonic processor (Scientz). The remaining debris was removed by centrifugation at 12 000 *g* at 4 °C for 10 min. Finally, the supernatant was collected, and the protein concentration was determined using a BCA kit according to the manufacturer's instructions.

### Trypsin Digestion

For digestion, the protein solution was reduced with 5 mm dithiothreitol for 45 min at room temperature in darkness and alkylated with10 mm 2‐chloroacetamide for 30 min at room temperature in darkness. Finally, trypsin was added at 1:50 trypsin‐to‐protein mass ratio for overnight digestion at 37 °C. 15 µL of each sample were used for proteomic analysis.

### Enrichment of Ubiquitinated Peptides

After enzyme digestion, the samples were reconstituted in 1.4 mL of pre‐cooled immunoaffinity purification (IAP) buffer, and pre‐treated anti‐K‐ε‐GG antibody beads (PTMScan ubiquitin remnant motif [K‐ε‐GG] Kit, Cell Signal Technology) were added. The mixture was incubated at 4 °C for 2 h, centrifuged at 2000 ×*g* for 3 min, and then discarded the supernatant. Anti‐K‐ε‐GG antibody beads were washed with 1 mL precooled IAP buffer for 3 times, and then washed with precooled water for 3 times. A volume (55 µL) of 0.15% TFA was added to the washed beads, incubated for 10 min at room temperature, added 0.15% TFA again, and centrifuged at 2000 ×*g* for 30 s. The supernatant was desalted using a commercial STAGE Tips desalting column (Thremo).

### LC‐MS/MS Analysis

The liquid chromatography‐tandem mass spectrometry (LC‐MS/MS) detection system consisted of a nanoflow high‐performance liquid chromatograph (HPLC) instrument (Easy nLC1200 System, Thermo) coupled to an Orbitrap Fusion mass spectrometer (Thermo) with a nanoelectrospray ion source (Thermo).

First, 1 µg of proteomic peptide mixture resolved in buffer A (0.1% formic acid (FA)) was loaded onto a 1 cm self‐packed trap column (ReproSil‐Pur C18‐AQ, 1.9 µm; Dr Maisch). The column temperature was maintained at 60 °C. Over a 90 min gradient (buffer A, 0.1% FA in water; buffer B, 0.08% FA in 80% ACN) at a flow rate of 600 nL min^−1^ (0–11 min, 5–10% buffer B; 11–91 min, 10–30% buffer B; 91–111 min, 30–45% buffer B; 111–112 min, 45–95% buffer B; and 112–120 min, 95% buffer B), the Orbitrap Fusion mass spectrometer (Thermo) was operated in the positive‐ion mode at an ion transfer tube temperature of 320 °C. The positive‐ion spray voltage was 2.2 kV. MS data acquisition in DIA mode was performed on an Orbitrap Q Exactive HF using 37 Fixed windows covering a mass range of 350–1400 m/z. The resolution was set to 60000 for MS1 and 30000 for MS2. The AGC was 3e6 in MS1 and 1e6 in MS2, with a maximum injection time of 20 ms in MS1 and 45 ms in MS2. NCE was set to 30%.

Next, 0.5 µg ubiquitinoomic peptide mixture dissolved in buffer A (0.1% formic acid (FA)) was loaded onto a 1‐cm self‐packed trap column (ReproSil‐Pur C18‐AQ, 1.9 µm; Dr Maisch). The column temperature was stably controlled at 60 °C. Over a 90 min gradient (buffer A, 0.1% FA in water; buffer B, 0.08% FA in 80% ACN) at a flow rate of 600 nL min^−1^ (0–4 min, 7–12% buffer B; 4–64 min, 12–30% buffer B; 64–84 min, 30–42% buffer B; 84–85 min, 42–95% buffer B; and 85–90 min, 95% buffer B), the Orbitrap Fusion mass spectrometer (Thermo) was operated in the positive‐ion mode at an ion transfer tube temperature of 320 °C. The positive‐ion spray voltage was 2.2 kV MS data acquisition in DIA mode was performed on Orbitrap Q Exactive HF using 33 Fixed windows covering a mass range of 400–1650 m/z. The resolution was set to 120000 for MS1 and 30000 for MS2. The AGC was 3e6 in both MS1 and MS2, with a maximum injection time of 60 ms in MS1 and auto in MS2. NCE was set to 27%.

### Spectronaut Analysis

In the analysis of HF data, search parameters of Spectronaut2 (version 18.0.230605.50606) were set as follows: mutation with NN predicted fragments to generate decoy; machine learning performed per run; precursor PEP cutoff 1; precursor q value cutoff 0.01; protein q value cutoff 0.01 at experiment level and 0.01 at run level; data filtering set to q value; cross‐run normalization True. In ubiquitinomic data analysis, the search settings were the same except for single hit definition and minor grouping on modified sequences but not stripped sequences, cross‐run normalization on, proteotypicity filter none, PTM localization on, and PTM probability cutoff set to 0.

### Single‐Cell RNA Sequencing Analysis

Mice were treated with control (*n* = 2) and tail‐suspended (*n* = 2) for 28 d. After that, tibiae and femurs were flushed with a 2 mL syringe using ice‐cold PBS to remove bone marrow cells. The tissues were enzymatically digested and dissociated into single cell for further single‐cell RNA sequencing analysis. A single cell 3 ′Library and Gel Bead Kit V3.1 (10x Genomics, 1000121) and Chromium Single Cell G Chip Kit (10x Genomics, 1000120) were loaded onto the Chromium single‐cell controller (10x Genomics) to generate single‐cell gel beads in the emulsion, where all generated cDNA shares a common 10x Barcode.

Single‐cell sequencing datasets were analyzed by package CellRanger (v6.0.0, 10× Genomics) and Seurat (v4.1.0).^[^ ^52^ ^]^ The 10× Genomics pre‐built mouse genome for mm10‐3.0.0 was used as reference. For quality control, genes expressed in fewer than 3 cells and gene count of less than 200 per cell were first excluded from datasets. Cells with UMI counts between 1000 and 40 000 were retained. Furthermore, cells exhibiting more than 10% mitochondrial‐associated genes and more than 0.2% red blood cell genes among their expressed genes were also removed. Putative doublets were filtered by DoubletFinder (v2.0.3).^[^ ^53^ ^]^

After normalizing the data with the NormalizeData, FindVariableFeatures was used to find highly variable genes, and all samples were integrated through the integrateData with the anchors found by the function FindIntegrationAnchors. ScaleData was utilized to scale the data, and principal component analysis (PCA) was performed on the dataset using RunPCA. For cell type annotation and monocyte subgroup classification, clustering and sub‐clustering were conducted using FindNeighbors (dims = 1:50) and the FindClusters (resolution = 1) functions followed by PCA dimension reduction on the most variable genes selected by FindVariableFeatures. RunUMAP (dims = 1:50) was used for dimensionality reduction and plotting UMAP maps. The FindAllMarkers function was utilized to identify marker genes. Each cell cluster was annotated manually based on known marker genes. Differential gene expression analysis was conducted using the FindMarkers function, employing the Wilcoxon rank sum test with a significance threshold of adjust p‐value < 0.05 and the absolute value of log2FC > 0.25. The gene ontology (GO) functional enrichment analysis was carried out using the clusterProfiler (v4.0.5)^[^ ^54^ ^]^ and EnrichMiner.^[^ ^27^ ^]^ Monocle2^[^ ^35^ ^]^ was used for single‐cell trajectory analysis of subpopulations of Monocyte.

### Metabolomics

Femurs and tibias form Ctrl and HLU male mice were placed in 1.5 mL Eppendorf tubes and added with 80% (v/v) HPLC methanol. They were then crushed with a grinder for 5 min, vortexed at 4 °C, and incubated at ‐80 °C for 1 h. Afterward, the samples were centrifuged at 4 °C at 14 000 g for 20 min using a refrigerated centrifuge, and the supernatant was transferred into new Eppendorf tubes. The supernatant was freeze‐dried into particles using a freeze‐drying machine (Beijing JM Technology Co., CV600) for further analysis with LC–MS (Waters I‐Class Binary Solvent Manager, USA) (Waters Vion IMS QTof, USA). For the cells processed by RCCS or control (*n* = 3), 2×10⁷ cells were collected and 500 µL of 80% (v/v) HPLC‐grade methanol was added. The cells were then lysed by sonication using an ultrasonic cell disruptor and incubated at ‐80 °C for 1 h. Subsequently, the samples were centrifuged at 14 000 *g* for 20 min at 4 °C in a refrigerated centrifuge (for the cell culture medium samples, starting from this step), and the supernatant was transferred to new Eppendorf tubes. The supernatant was freeze‐dried into particles using a freeze‐drying machine for further analysis by LC‐MS. Raw data generated by mass spectrometry were qualitatively and quantitatively analyzed using Progenesis QI 24.78.090 (Waters UK). Default parameters were used for the search conditions, and the search database was HMDB (20230320) LIPIDMAPS.

### Metabolism Data Preprocess

The resulting feature tables were processed by R and MetaboAnalyst for subsequent analysis. The data were pretreated using the “80% rule”^[^ ^55^ ^]^ to reduce the missing value input. For the tissue data with QC samples, the features with relative standard deviations (RSDs) > 30% were removed from all the QC samples. The pretreated data were auto‐scaled, then analyzed using principal component analysis (PCA) and orthogonal partial least squared discriminant analysis (OPLS‐DA).^[^ ^56^ ^]^ The features with variable importance in the projection (VIP) > 1 and *p*‐value < 0.05 were considered to be differential compounds. Metabolic enrichment analysis was performed by MetaboAnalyst 5.0.^[^ ^57^ ^]^

### Data Normalization and Differential Expression

For both the peptide and protein MS data sets, proteins/peptides with more than two (protein) or three (peptide) missing detections in any set of four replicate samples were excluded. The filtered data were then quantile normalized and log2 transformed for further analysis. Missing values were randomly replaced based on the small values captured in each sample.^[^ ^58^ ^]^ Normalized and scaled data were subjected to differential expression analysis using Limma R package.^[^ ^59^ ^]^ Proteins with P value < 0.05 and Fold Change > 1.5 were considered as significantly differentially expressed between two groups. KEGG pathway enrichment analysis of differential protein was carried out using clusterProfiler (v4.0.5). For the peptide MS data sets, the Spearman correlation between each peptide and its corresponding protein was calculated to remove the differentially ubiquitinated peptides which have a higher positive correlation with the differentially expressed protein. The retained peptide segments were considered to have real difference in the ubiquitination data. For those differential peptides who have an opposite trend of expression difference compared to their corresponding proteins were considered to be degrading ubiquitination modifications, while others were considered to be non‐degrading ubiquitination modifications. The GSEA analysis of differential peptides was calculated by the EnrichMiner. UbiBrowser2.0^[^ ^29^ ^]^ was used to predict the ubiquitination substrate for a given enzyme in the proteome data. All computations were performed in R (version 4.2.3). The materials for the omics process model diagram are provided by Figdraw.

### Patient Serum Analysis

10 elderly men with osteoporosis (OP) and 10 elderly men with normal bone mineral density (BMD) were included. Participants with tumors or severe liver disease were excluded. The diagnosis of OP was defined according to the WHO criteria, with BMD measurements using dual‐energy X‐ray absorptiometry (DXA) scans (GE‐UNAR Company, Boston, MA, USA [coefficient of variation, 1.2%]) by the same trained operator. The study was approved by the Ethics Committee of the Chinese PLA General Hospital (No. S2021‐094‐01).

Fasting blood was obtained to test glutamine levels using the Glutamine/Glutamate‐Glo Assay (Promega, J8021) according to the manufacturer's instruction. And bone metabolism, gonad and thyroid hormone levels were measured, including testosterone (T), estrogen (E), osteocalcin (OC), procollagen type 1 N‐prepeptide (P1NP), parathyroid hormonealanine (PTH), C‐terminal telopeptide of type 1 collagen (CTX), vitamin D (vit D) and triglyceridethyroid stimulating hormone (TSH). Other common clinical markers were also measured, including creatine kinase (CK), creatine kinase isoenzyme MB (CK‐MB), lactate dehydrogenase (LDH), homocysteine (HCY), aminotransferase (ALT), aspartate aminotransferase (AST), creatinine, fasting plasma glucose (FPG), albumin(Alb), low‐density lipoprotein cholesterol (LDL‐C), total cholesterol (TC), serum uric acid (SUA), erythrocyte sedimentation rate (ESR), glycosylated hemoglobin (HbA1c), hemoglobin (Hb), and lymphocyte count (LYC) on Beckman automatic analyzer in the clinical lab. Estimated glomerular filtration rate (eGFR) was calculated by creatinine related international formula.^[^ ^60^ ^]^

### Statistical Analysis

For patient serum study, continuous variables were expressed as mean ± standard deviation (SD), and categorical variables were presented as number with percentages. Student's t‐test and the chi‐square test was applied to analyze the differences between OP and control groups. Pearson correlation analysis was performed to determine the correlation between Gln and clinical features, and receiver operating characteristic (ROC) curves were further used to compare differences in the predictive power for OP. For other studies, statistical analyses were performed using GraphPad Prism (v8.0.1) software for statistical significance. The p‐value was determined by the Student's t‐test for two‐group or one‐way ANOVA test for multiple‐group and P values <0.05 were considered statistically significant.

### Data Availability

The single cell RNA‐seq datasets have been submitted to the NCBI database (https://www.ncbi.nlm.nih.gov/geo/query/acc.cgi?acc=GSE263705) under the accession number GSE263705. The mass spectrometry proteome, ubiquitinomes and Metabolomes data generated in this study have been deposited to the ProteomeXchange Consortium (https://proteomecentral.proteomexchange.org) via the iProX partner repository with the dataset identifier PXD050890 and PXD064760. Source data are provided with this paper.

## Conflict of Interest

The authors declare no conflict of interest.

## Author Contributions

Y.D., F.T., M.L., and P.Y. contributed equally to this work. C.P.C., L.Q.Z., W.J.S., and F.H. conceptualized the study. Y.D. and F.T. designed and conducted biochemical experiments and cell biology experiments. Y.D. and F.T. established the animal model. J.Z., F.H., F.T., and M.Q.L. collected samples and analyzed immunohistochemistry. Y.H.W., F.T., C.C., and Y.J.Z. performed MS experiments. W.J.S., F.H., P.Y., J.Z., D.L., and C.C. conducted bioinformatics and statistical analysis. C.P.C. and F.H. drafted the original manuscript. Writing—review & editing were done by W.J.S., F.H., and L.Q.Z.

## Supporting information



Supporting Information

Supplemental Table 1

Supplemental Table 2

Supplemental Table 3

Supplemental Table 4

Supplemental Table 5

Supplemental Table 6

Supplemental Table 7

Supplemental Table 8

## Data Availability

The data that support the findings of this study are available in the Supporting Information of this article.
