# Supplementary material for: Integrative Omics Reveals Glutamine Catabolism‐Driven Apoptotic Suppression in Monocytes upon Mechanical Unloading
Source: Adv Sci (Weinh). 2025 Aug 18;12(42):e00585. doi: 10.1002/advs.202500585 (PMC12622456; doi:10.1002/advs.202500585)
Supplement: Supplementary file 1 — Supporting Information [file ADVS-12-e00585-s006.docx]

Supporting Information

Integrative Omics Reveals Glutamine Catabolism-Driven Apoptotic Suppression in Monocytes upon Mechanical Unloading

Yi Ding, Fan Tong, Mingqiu Liu, Pin Yang, Jing Zeng, Yinghua Wei, Chunlin Li, Dong Li, Cheng Chang, Yangjun Zhang, Shaoqiong Yi, Fan Hu*, Wenjie Shu*, Lingqiang Zhang*, Chun-Ping Cui*


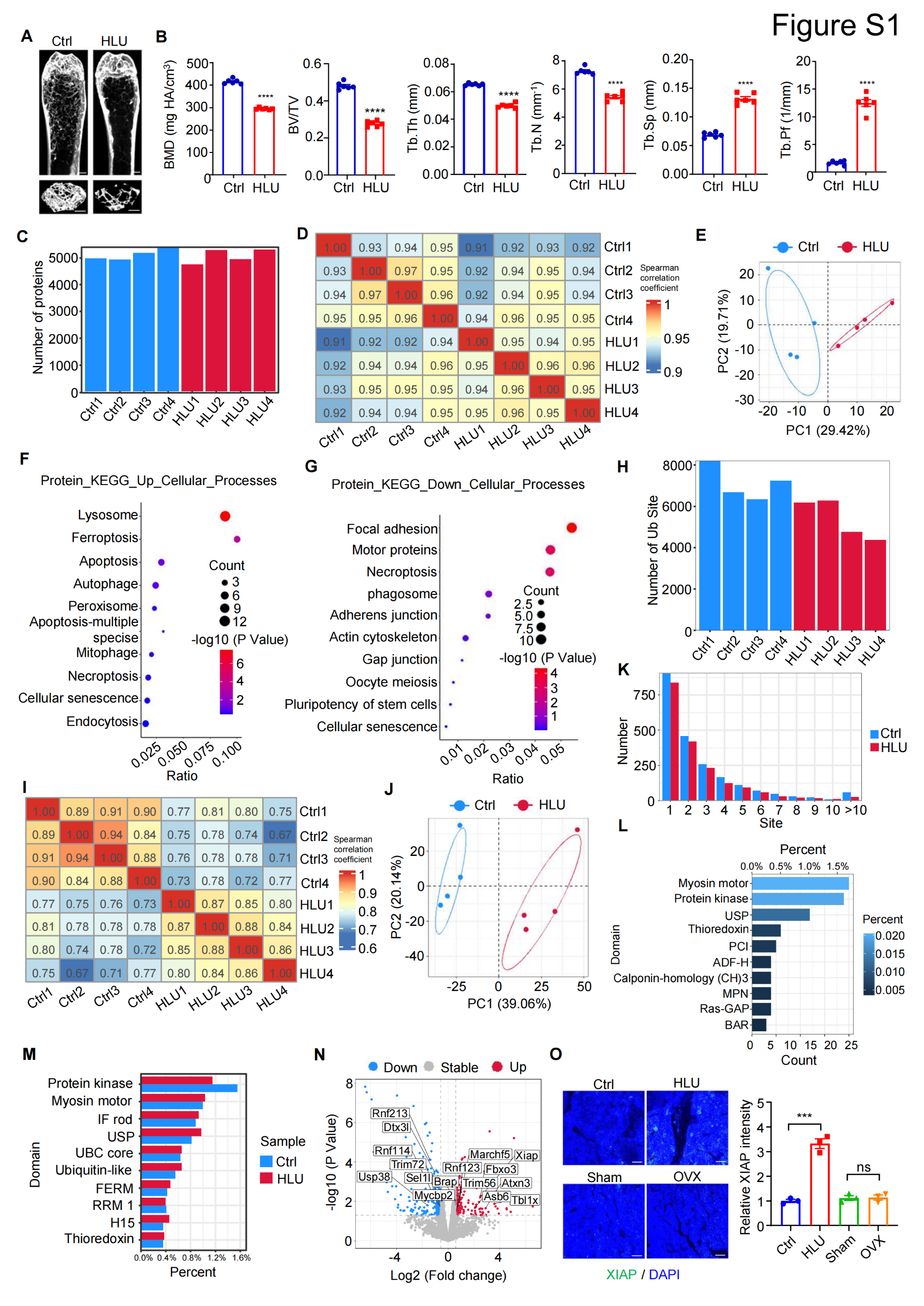


**Supplemental Figure 1. Integrative proteomics revealed ubiquitylation patterns in bone tissue upon reduced mechanical force.**

(A). Representative Micro-CT reconstruction images of whole femoral (top) and trabecular (bottom) bones from Ctrl and HLU for 28 days mice. n = 6 per group. Scale bars, 0.5 mm.

(B). Quantitative Micro-CT analyses of distal femurs from Ctrl and HLU mice, including bone mass density (BMD), bone volume per tissue volume (BV/TV), trabecular thickness (Tb.Th), trabecular number (Tb.N), trabecular spacing (Tb.Sp) and trabecular pattern factor (Tb.Pf). n = 6 per group.

(C). Histogram of the captured protein number in each sample. Red: HLU group; Blue: control group. n = 4 per group.

(D). Heatmap illustrating the Spearman correlation of eight proteome samples. Red indicates positive correlation, and blue indicates negative correlation. n = 4 per group.

(E). Principal Component Analysis (PCA) plot showing separation between Ctrl (blue) and HLU (red) samples in proteome data. Blue and red ovals represent confidence ellipses of Ctrl and HLU groups. n = 4 per group.

(F, G). Dot plot showing the pathways which belong to the KEGG Cellular Processes category enriched by genes corresponding to up-regulated (F) or down-regulated (G) differentially expressed proteins. The size of the point indicates the count of proteins that enriched by genes corresponding. The color of the point indicates the P values. n = 4 per group.

(H). Histogram showing the number of di-GG sites captured in each sample. Red: HLU group; Blue: control group. n = 4 per group.

(I). Heatmap illustrating the Spearman correlation of eight ubiquitome samples. Red indicates positive correlation, and blue indicates negative correlation. n = 4 per group.

(J). PCA plot showing separation between Ctrl (blue) and HLU (red) samples in ubiquitome data. Blue and red ovals represent confidence ellipses of Ctrl and HLU groups. n = 4 per group.

(K). Column chart showing the distribution of ubiquitin sites identified in bone tissues from Ctrl (blue) and HLU (red) mice. n = 4 per group.

(L). Histogram showing the numbers and proportions (color) of the top 10 structural domains where differential ubiquitination modification sites are located. n = 4 per group.

(M). Column chart illustrating the top 10 structural domains for comparison between the Ctrl (blue) and HLU (red) where ubiquitination modification sites are located. n = 4 per group.

(N). Volcano plot showing up-regulated (red) and down-regulated (blue) differentially expressed proteins in HLU mice compared to Ctrl mice. Differentially expressed proteins of ubiquitin-conjugating enzyme (E2), ubiquitin ligase (E3), and deubiquitinating enzyme (DUB) are highlighted. n = 4 per group.

(O). Immunofluorescence staining to detect XIAP (green) expression in bone samples from Ctrl and HLU female mice (top), and sham-operated and OVX female mice (bottom). Nuclei were counterstained with DAPI. Scale bars: 50 μm. Quantitative analysis of XIAP expression was conducted using ImageJ (right). n = 3 per group.

Data are shown as the mean ± SEM and compared using two-tailed Student’s t-test. ***P < 0.001, ****P < 0.0001, n.s., not significant.

**
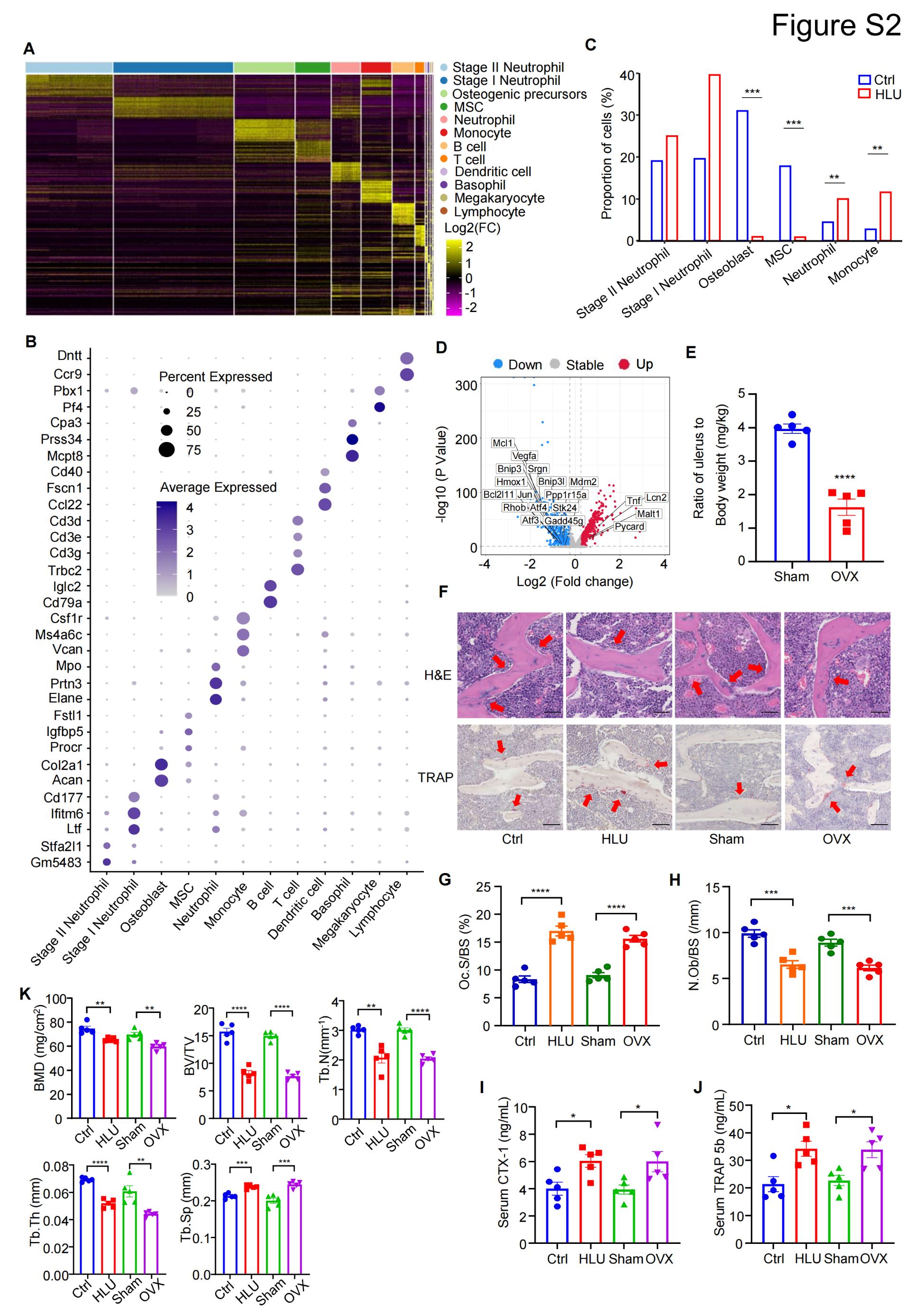
**

**Supplemental Figure 2. Single-cell RNA sequencing data revealed significant changes in apoptosis-related genes.**

(A). Heatmap showing the top 20 highly variable genes of each cell type. n = 2 per group.

(B). Dot plot showing the average expression levels of marker genes for each cell type. The size of the point indicates the proportion of cells that highly express a gene in this cell cluster. The color of the point indicates the level of expression of a gene in this cell cluster. n = 2 per group.

(C). Bar chart displaying the average proportion of each cell type in the two groups of samples. Data are mean ± SD. n = 2 per group.

(D). Volcano plot showing up-regulated (red) and down-regulated (blue) differentially expressed genes in monocytes of HLU mice compared to Ctrl mice in single-cell data. Apoptosis-related genes of monocyte between Ctrl and HLU are highlighted. n = 2 per group.

(E). Histomorphometric evaluation of the uterine weight-to-body weight ratio (uterosomatic index) in Sham-operated versus ovariectomized (OVX) female C57BL/6 mice at 4 weeks post-surgery. n = 5 per group.

(F). Representative images of TRAP staining for quantifying osteoclasts and H&E staining for the quantifying of osteoblasts in Ctrl, HLU, Sham and OVX mice. Scale bars, 50 μm. n = 5 per group.

(G, H). Histological analysis the osteoclasts surface per unit of trabecular bone surface (Oc.S/BS) (G) and the number of osteoblasts per unit of trabecular bone surface (N.Ob/BS) (H) in Ctrl, HLU, Sham and OVX mice. n = 5 per group.

1. . Mouse serum CTX-1 were detected by ELISA. n= 5 per group.
2. . Mouse serum TRAP 5b were detected by ELISA. n= 5 per group.
3. Histomorphometric analysis of trabecular bones, including bone mass density (BMD), bone volume per tissue volume (BV/TV), trabecular number (Tb.N), trabecular thickness (Tb.Th) and trabecular spacing (Tb.Sp) from Ctrl, HLU and Sham-operated and OVX female mice. n = 5 per group.

Data are mean ± SEM and compared using two-tailed Student’s t-test. n = 3 per group. *P < 0.05, **P<0.01, ***P < 0.001, n.s., not significant.

**
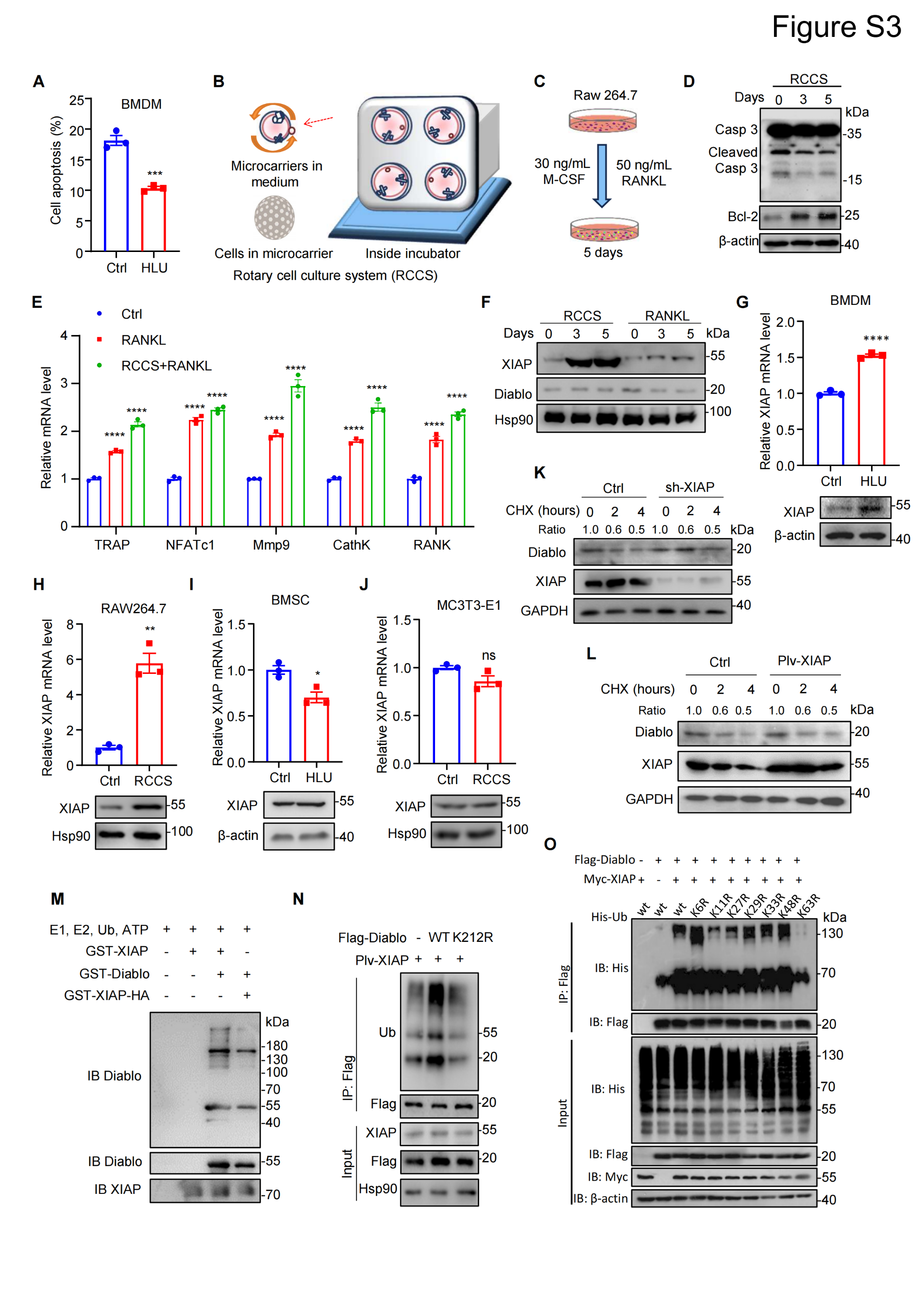
**

**Supplemental Figure 3. Unloading exposure promoted the expression of XIAP in the osteoclast lineage cells.**

(A). BMDM cells were isolated from Ctrl and HLU male mice followed by the assessment of relative cell apoptosis using flow cytometry. n = 3 per group.

(B). Schematic representation of rotary cell culture system (RCCS).

(C). Schematic illustrated the dosage of RANKL and M-CSF-induced RAW264.7 cell differentiation.

(D). Immunoblot of apoptosis-related proteins in RAW264.7 with RCCS culture. n = 3 per group.

(E). Quantitative RT-PCR analysis was conducted to measure the relative TRAP, NFATc1, Mmp9, CathK and RANK mRNA levels in RAW264.7 cells after RCCS treatment (the RCCS+RANKL group in the panel) or control treatment (the RANKL group in the panel) for 3 days, followed by culturing cells for 5 days with 30 ng/mL M-CSF and 50 ng/mL RANKL. The Ctrl group indicates no RCCS treatment and no RANKL treatment. n = 3 per group.

(F). Immunoblot analysis of XIAP and Diablo in RAW264.7 cells following treatment with RCCS or RANKL for 0, 3, and 5 days. n = 3 per group.

(G-J). Quantitative RT-PCR and immunoblot analysis of XIAP mRNA and protein in BMDM cells from Ctrl and HLU mice (G), RAW264.7 cells after treatment with RCCS for 0 and 5 days (H), BMSC from Ctrl and HLU mice (I) and MC3T3-E1 cells after RCCS treatment for 0 and 5 days (J). n = 3 per group.

(K, L). RAW264.7 cells with XIAP knockdown XIAP (K) or stably expressing XIAP (L) were subjected to increasing durations of CHX treatment (0 hour, 2 hours, 4 hours). Following treatment, protein lysates were analyzed via immunoblotting to determine the levels of XIAP and Diablo. n = 3 per group.

(M). In vitro ubiquitination assay. Purified GST-XIAP or GST-XIAP H466A and GST-Diablo proteins were incubated with E1, E2, Ub and ATP. Reactions were performed as described in the Materials and Methods section. Samples were analyzed by western blotting with indicated antibodies. n = 3 per group.

(N). RAW264.7 cells overexpressing Diablo WT or K212R mutant were stably transfected with XIAP, and the cell lysates were collected for IP and immunoblot analysis. n = 3 per group.

(O). Diablo ubiquitylation linkage assays were performed by co-transfecting His-ubiquitin wild type (WT) or different ubiquition modificated sites mutants with Flag-Diablo and Myc-XIAP in HEK293T cells. Cell lysates were subsequently collected for immunoprecipitation (IP) and immunoblot analysis. n = 3 per group.

All data are mean ± SEM and compared using two-tailed Student’s t-test. *P < 0.05, **P<0.01, ***P < 0.001, ****P < 0.0001, n.s., not significant.

**
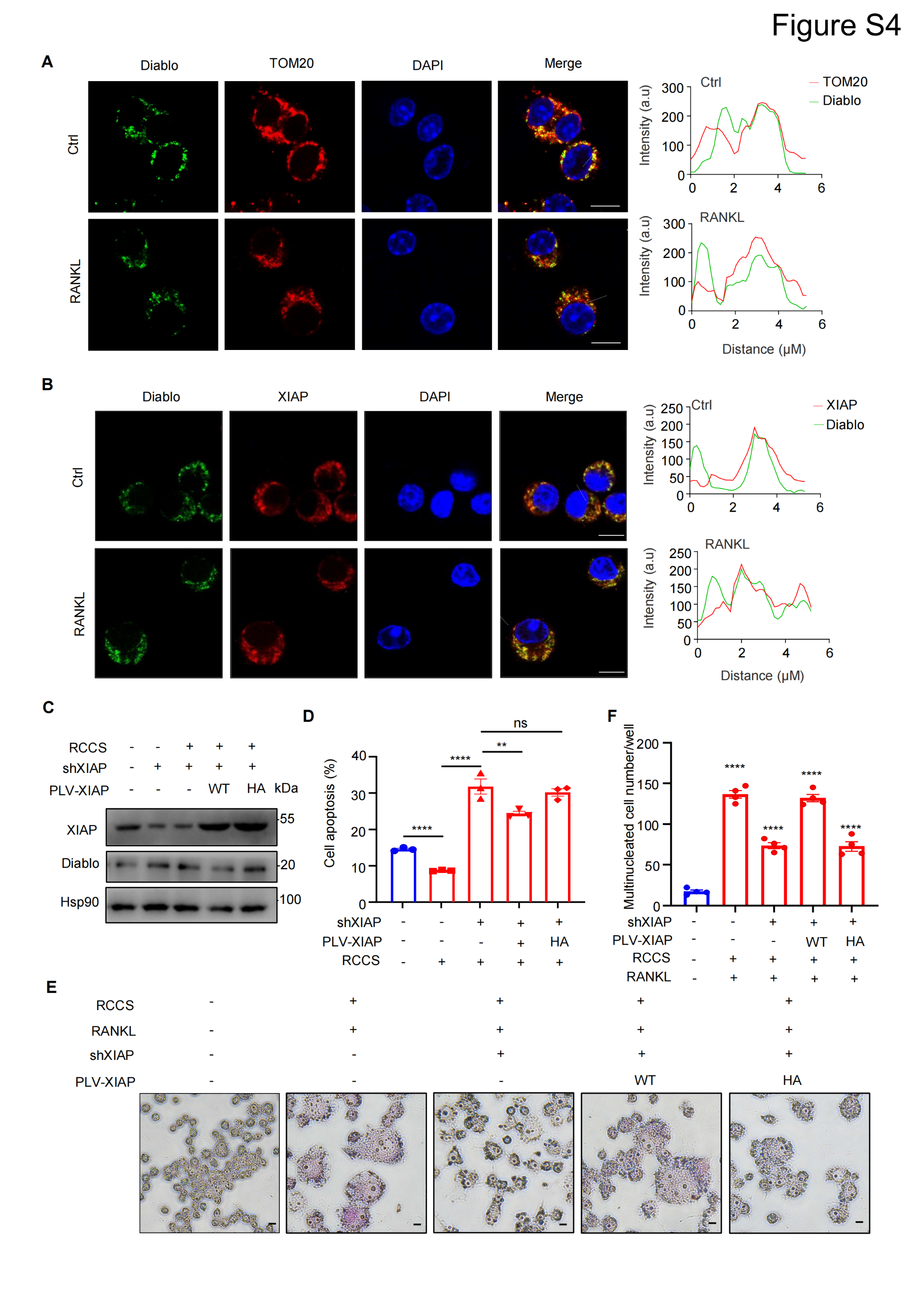
**

**Supplemental Figure 4. RCCS stimulation, rather than RANKL induction, enhanced the colocalization of XIAP and Diablo in mitochondria.**

(A, B). Immunofluorescence and quantitative analysis were conducted to evaluate the subcellular localization of Diablo (green) and TOM (red) (A), or the localization of Diablo (green) with XIAP (red) (B) in RAW264.7 cells treated with RANKL for 0 and 5 days. Confocal microscopy images are displayed (left), scale bar, 10 μm. Fluorescence intensity was quantified (right). n = 3 per group.

(C). Immunoblot analysis of Diablo in XIAP-silenced RAW264.7 cells that were stably transfected with negative control, XIAP WT or XIAP H466A mutant, treated with RCCS for 5 days. n = 3 per group.

(D). The RAW264.7 cells with XIAP knockdown were stably overexpressed XIAP WT or H466A mutant. Cells were cultured with RCCS for 5 days, Annexin V assay was conducted to examine the percentage of apoptotic cells. n = 3 per group.

(E-F). XIAP-knockdown RAW264.7 cells were stably transfected to overexpress either wild-type XIAP (XIAP WT) or the H466A mutant. Following either RCCS treatment or control treatment for 3 days, and subsequent culture for 5 days with 30 ng/mL M-CSF and 50 ng/mL RANKL, representative images of TRAP staining in RAW264.7 cells are shown (E), Scale bars: 10 μm. The number of TRAP-positive multinucleated cells was quantified and is presented in the histogram (F). n = 4 per group.

All data are mean ± SEM and compared using two-tailed Student’s t-test or ANOVA. *P < 0.05, **P<0.01, ***P < 0.001, ****P < 0.0001, n.s., not significant.


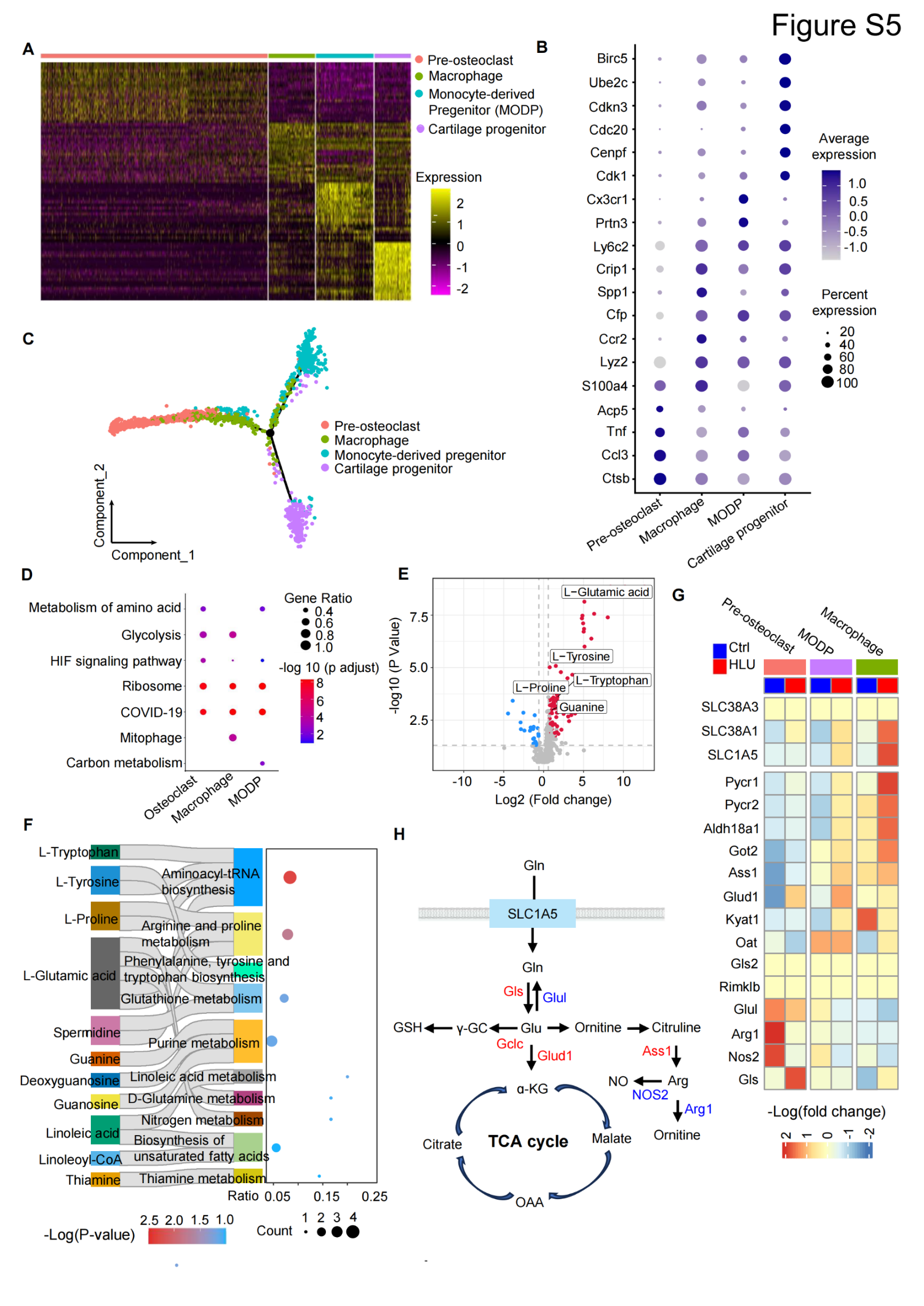


**Supplemental Figure 5. Mechanical unloading promotes Gln metabolism in osteoclast lineage cells.**

(A). Heatmap showing the top 20 highly variable genes of each monocyte subgroup. n = 2 per group.

(B). Dot plot showing the scaled average expression levels of marker genes for each monocyte subgroup. The size of the point indicates the proportion of cells that highly express a gene in cell subclusters. The color of the point indicates the level of expression of a gene in cell subclusters. n = 2 per group.

(C). Differentiation trajectories of monocytes, grouped based on subgroups. n = 2 per group.

(D). KEGG pathway enrichment analysis of down-regulated genes in osteoclast, macrophage and monocyte-derived progenitor (MODP). The size of the point indicates the gene ratio in cell subclusters. The color of the point indicates the P values. n = 2 per group.

(E). Volcano plot showing up-regulated (red) and down-regulated (blue) differentially expressed metabolites in HLU mice compared to Ctrl mice in the negative mode, with the primary metabolites in KEGG enrichment labeled. n = 2 per group.

(F). Metabolic KEGG enrichment analysis with corresponding differential metabolites. The size of the point indicates the count of metabolites in enriched pathways. The color of the point indicates P value. Control, n = 2. HLU, n = 3.

(G). Heatmap showing the scaled average expression of genes related to glutamine (Gln) metabolism across monocyte subclusters. n = 2 per group.

(H). Pathway of Gln metabolism, red fonts represent up-regulated genes in monocyte and blue represent down-regulated gene


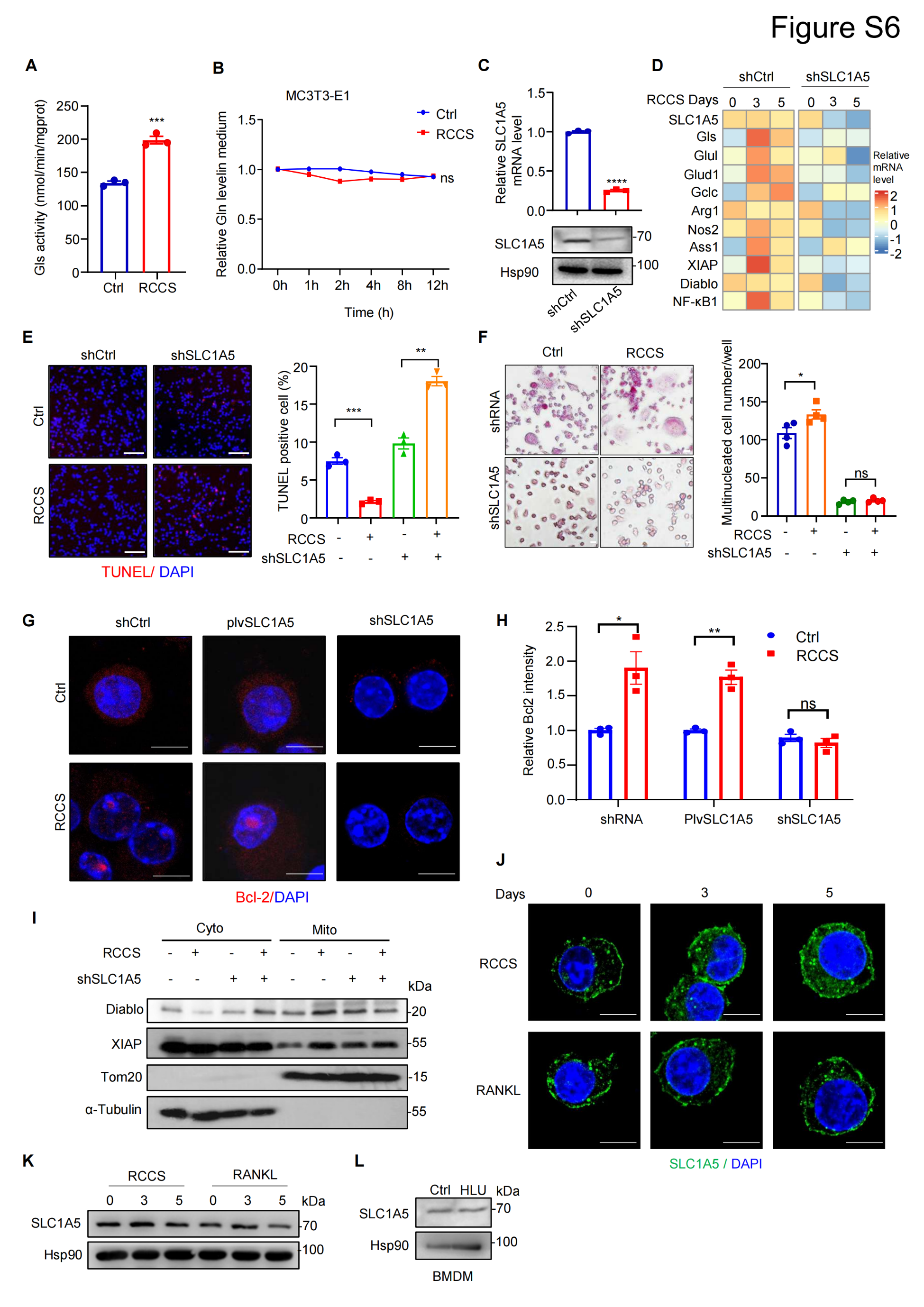


**Supplemental Figure 6. SLC1A5-mediumted Gln uptake is essential for the survival of osteoclast lineage cells during mechanical unloading.**

(A). Glutaminase (Gls) activity after RCCS treatment of RAW264.7 cells are depicted on the histograms. n = 3 per group.

(B). MC3T3-E1 cells were treated with RCCS for 0 to 12 hours, and the uptake of Gln was assessed by measuring the remaining Gln in the medium. n = 4 per group.

(C). Quantitative RT-PCR and Immunoblot analysis were performed to assess the levels of SLC1A5 mRNA and protein in RAW264.7 cells with either SLC1A5 knockdown or negative control. n = 3 per group.

(D). Quantitative RT-PCR analysis the mRNA levels of indicated genes in knockdown SLC1A5 and negative control of RAW264.7 after RCCS treatment for 0, 3 and 5 days. n = 3 per group.

(E). Representative images of SLC1A5 knockdown or negative control RAW264.7 cells after 0 and 5 days of RCCS treatment were detected by TUNEL (red) staining (left). Scale, 50 μm. Quantitative analysis was performed by ImageJ (right). n = 3 per group.

(F). Representative images of TRAP staining of SLC1A5 knockdown or negative control RAW264.7 cells after 3 days of RCCS treatment, followed by culturing cells for 5 days with 30 ng/mL M-CSF and 50 ng/mL RANKL. Scale bar, 10 μm. TRAP-positive multinucleated cells were then counted, and the results are presented as a histogram (right). n = 4 per group.

(G-H). RAW264.7 cell lines with stable SLC1A5 knockdown, negative control or SLC1A5 overexpression were subjected to RCCS or control treatments. Fluorescence quantitative analysis of Bcl-2 was performed on the images (H). n = 3 per group.

(I). SLC1A5 was silenced in RAW264.7 cells using shRNA. The transfected cells were then cultured in RCCS for 3 days. Subsequently, mitochondria and cytoplasm were isolated, and the expression of XIAP and Diablo in these compartments was determined by western blot analysis. n = 3 per group.

(J). Representative images of SLC1A5 IF staining in RAW264.7 cells treated with RCCS or RANKL for indicated times. Scale, 10 μm. n = 3 per group.

(K). Immunoblot analysis of SLC1A5 in RAW264.7 cultured with RCCS or RANKL treatment for indicated times. n = 3 per group.

(L). Immunoblot analysis of SLC1A5 in bone tissues from HLU or control mice.

Data are shown as the mean ± SEM. **P<0.01, ***P < 0.001, ****P < 0.0001, n.s., not significant. Statistical differences were determined using Student’s t-test.

**
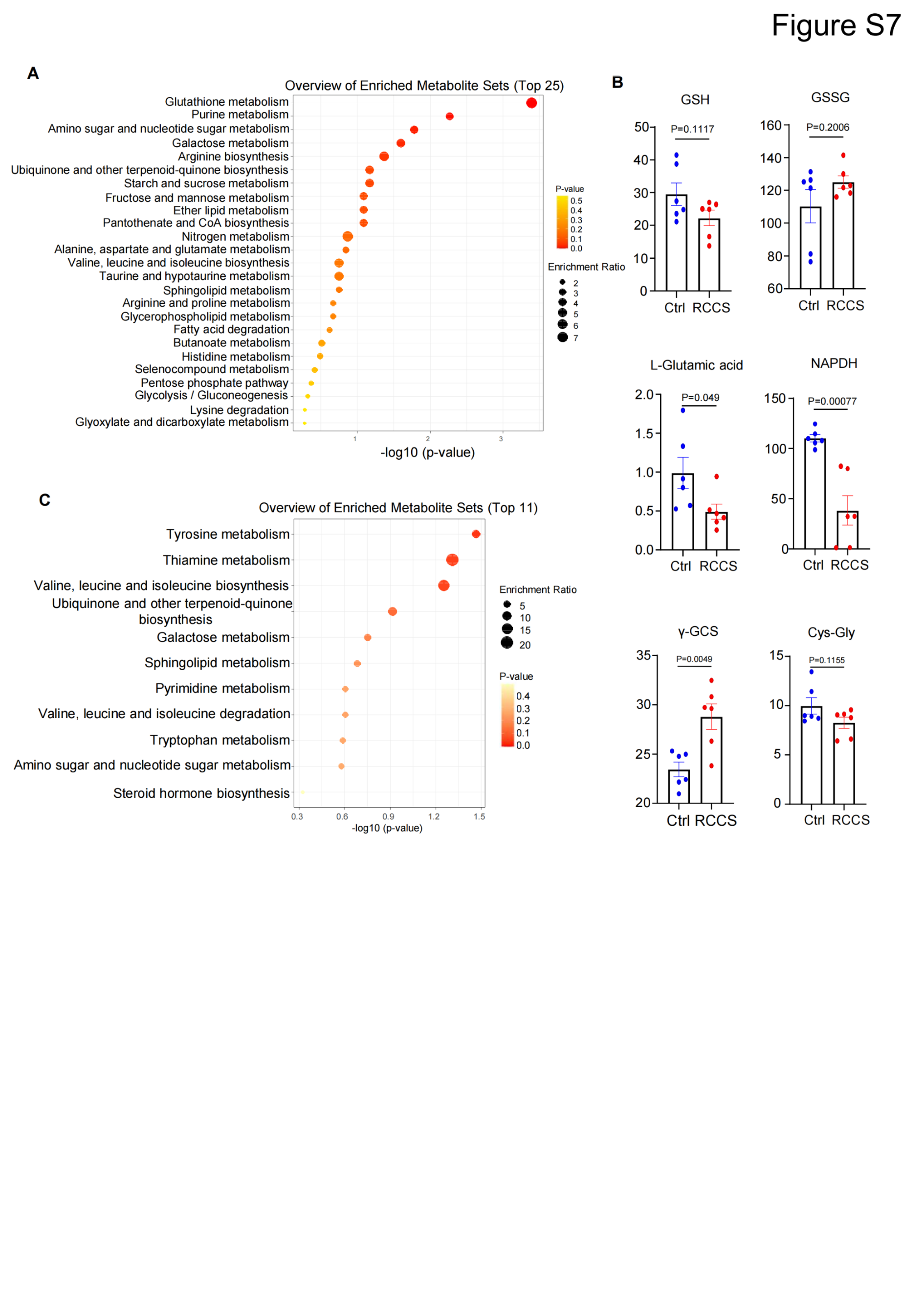
**

**Supplemental Figure 7. RCCS treatment causes changes in GSH metabolism in RAW264.7 cells.**

(A). Illustrates the top 25 KEGG metabolic pathways enriched with differential metabolites between the RCCS and control cell lines, obtained using MetaboAnalyst. P values are shown by two-way ANOVA with Sidak’s multiple comparison test. The size of the point indicates the enrichment ratio. The color of the point indicates the P values. n =3 per group.

(B). Relative abundance of glutathione (GSH), glutathione oxydized (GSSG), L-glutamic acid, NADPH, γ-glutamate cysteine ligase (γ-GCS) and Cysteinylglycine (Cys-Gly) from control and RCCS cells, determined by LC–MS analysis, Data represents mean ± SEM, n =3 per group. Statistical differences were determined using Student’s t-test.

(C). Illustrates the top 11 KEGG metabolic pathways enriched with differential metabolites between the RCCS and control culture media, obtained using MetaboAnalyst. P values are shown by one-way ANOVA with Sidak’s multiple comparison test. The size of the point indicates the enrichment ratio. The color of the point indicates the P values. n =3 per group.


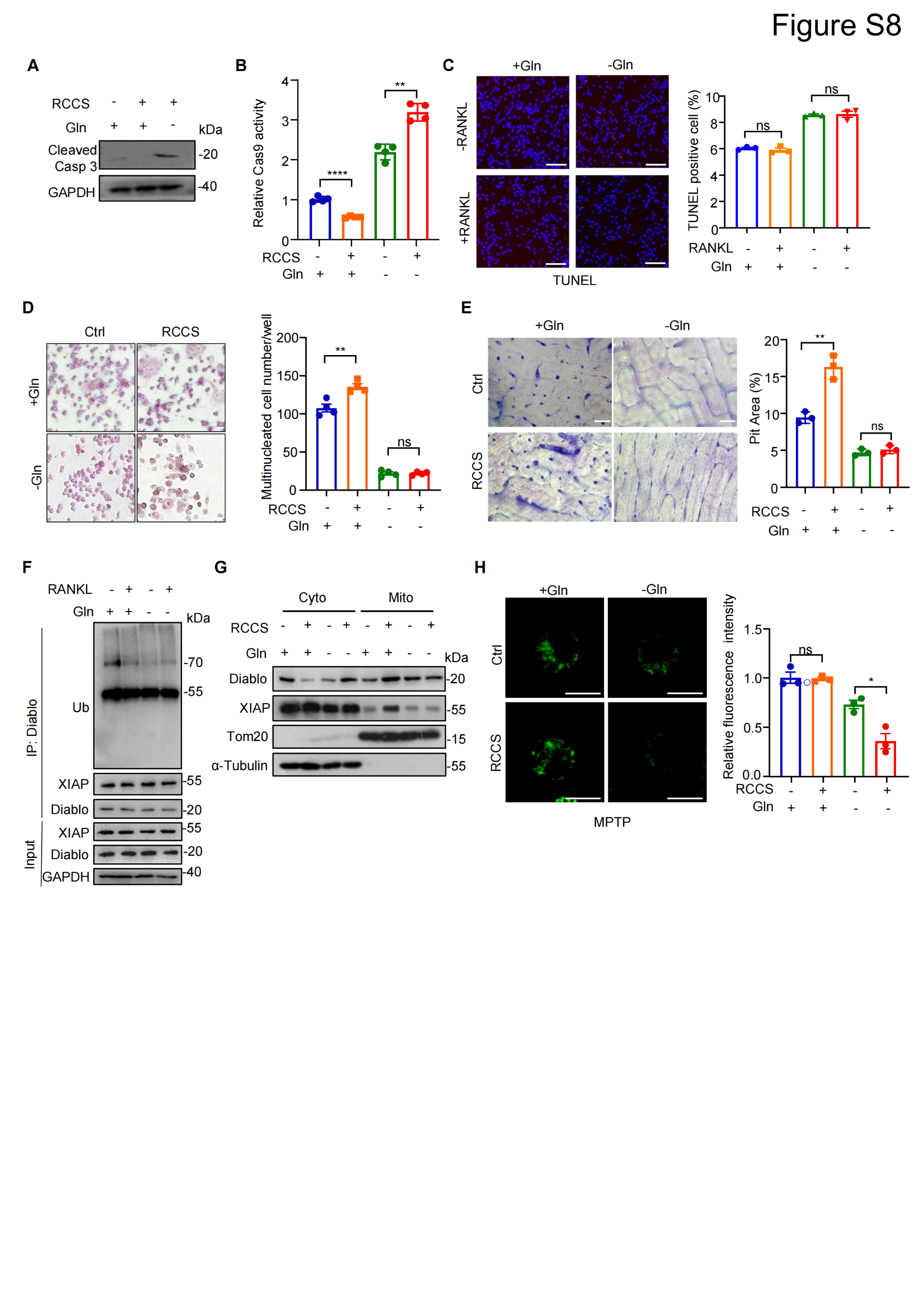


**Supplemental Figure 8. During RANKL-induced osteoclastogenesis, cell apoptosis remains unchanged in cells deprived of Gln.**

(A). RAW264.7 cells were treated with RCCS in normal or glutamine-deprivation medium conditions for 3 days. Cleaved caspase-3 expression was subsequently analyzed using western blotting. n = 3 per group.

(B). Caspase 9 activity was evaluated in RAW264.7 cells treated with RCCS or control for 3 days, under normal or glutamine-deprivation medium conditions. n = 4 per group.

(C). Representative images of apoptosis induced by RANKL in RAW264.7 cells were observed over 0, 3, and 5 days in both normal or Gln-deprivation medium using TUNEL staining. Quantitative analysis was performed using ImageJ. Scale bar, 50 μm. n = 3 per group.

(D). Representative images of TRAP staining in RAW264.7 cells treated with RCCS 3 days in normal or Gln-deprivation medium, followed by culturing cells for 5 days with 30 ng/mL M-CSF and 50 ng/mL RANKL. Scale bar, 10 μm. TRAP-positive multinucleated cells were then counted, and the results are presented as a histogram (right). n = 4 per group.

(E). Resorption pit assay illustrating the effects of RCCS and glutamine (Gln) on the bone resorption of bovine bone slices in vitro. Scale bar, 100 μm. Quantitative analysis of pit area was performed by ImageJ (right). n = 3 per group.

(F). Immunoprecipitates (IP) of Diablo ubiquitination in RAW264.7 cells in normal medium or Gln-deprivation medium after 0 and 5 days with RANKL treatment. n = 3 per group.

(G). RAW264.7 cells following RCCS treatment in normal medium or Gln-deprivation DMEM medium. Subsequently, mitochondria and cytoplasm were isolated, and the expression of XIAP and Diablo in these compartments was determined by western blot analysis. n = 3 per group.

(H). Representative images of mitochondrial permeability transition pore (MPTP) staining in RAW264.7 cells treated with RCCS or control for 3 days are shown, conducted in normal or Gln-deprivation DMEM medium. The relative fluorescence intensity is quantified in the histogram (right). n = 3 per group.

Data are shown as the mean ± SEM and were compared using the one-way ANOVA. **P<0.01, ***P < 0.001, ****P < 0.0001, n.s., not significant.


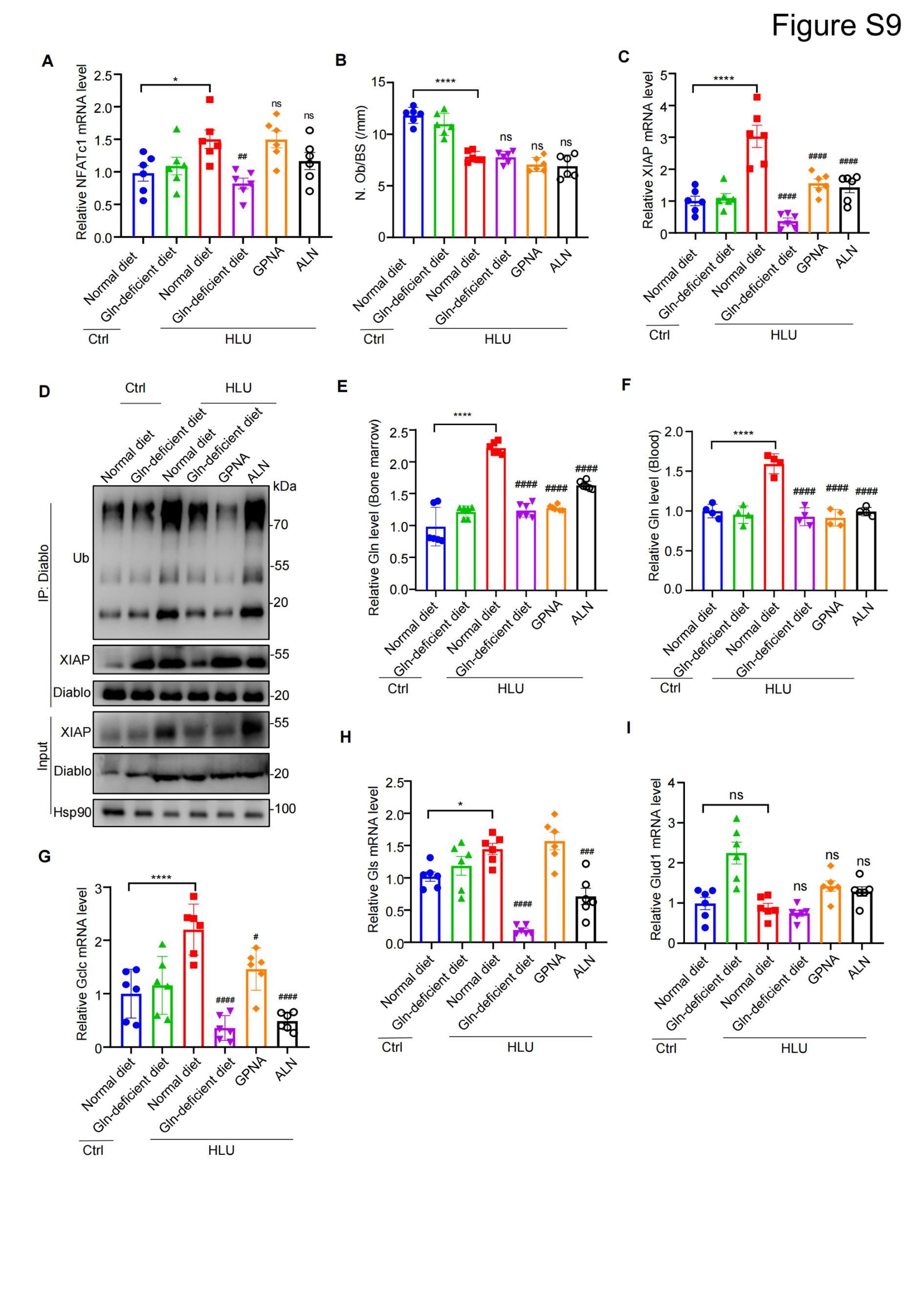


**Supplemental Figure 9. Targeting the Gln/SLC1A5 axis effectively preserved bone mass under mechanical unloading.**

(A). Quantitative RT-PCR analysis of relative mRNA levels of NFATc1 in bone tissues from different treatment group. n = 6 per group.

(B). Histological analysis the number of osteoblasts per unit of trabecular bone surface (N Ob/BS) of femurs from different treatment mice. n = 6 per group.

(C). Quantitative RT-PCR analysis of the relative mRNA levels of XIAP of bone tissues from different treatment mice. n = 6 per group.

(D). IP of Diablo ubiquitination in bone tissue from different treatment mice. n = 3 per group.

(E). Relative Gln content in bone marrow cells from different treatment mice. n = 6 per group.

(F). Relative Gln content in serum from different treatment mice. n = 4 per group.

(G-I). Quantitative RT-PCR analysis of mRNA levels of Gclc (G), Gls (H) and Glud1 (I) of bone tissue from different treatment mice. n = 6 per group.

Data are shown as the mean ± SEM, and were compared using the one-way ANOVA. *P < 0.05, ***P < 0.001, ****P < 0.0001. #P < 0.05, ##P<0.01, ###P < 0.001, ####P < 0.0001 versus HLU with normal diet. n.s., not significant.

**Supplementary Tables**

Table S1. Degrading ubiquitination Enrichminer.

Table S2. Non-degrading ubiquitination Enrichminer.

Table S3. Monocyte DEG Enrichminer.

Table S4. Characteristic of participants.

Table S5. Antibodies.

Table S6. Plasmids kept in lab or constructed in this study.

Table S7. The qPCR primers used in this study.

Table S8. Sequences for shRNA.
