# Supplementary material for: Integrative Omics Reveals Glutamine Catabolism‐Driven Apoptotic Suppression in Monocytes upon Mechanical Unloading
Source: Adv Sci (Weinh). 2025 Aug 18;12(42):e00585. doi: 10.1002/advs.202500585 (PMC12622456; doi:10.1002/advs.202500585)
Supplement: Supplementary file 5 — Supplemental Table 4 [file ADVS-12-e00585-s007.docx]

Table S4. Characteristic of participants.

|  | Normal  (N = 10) | OP  (N = 10) | *P* value |
| --- | --- | --- | --- |
| Age, mean ± SD (years) | 80.4 ± 3.78 | 82.10 ± 3.76 | 0.326 |
| BMI, mean ± SD (kg/m^2^) | 23.79 ± 2.23 | 22.57 ± 1.59 | 0.174 |
| SBP, mean ± SD (mmHg) | 138 ± 16 | 137 ± 14 | 0.800 |
| DBP, mean ± SD (mmHg) | 77 ± 13 | 77 ± 7 | 0.200 |
| T2DM, n (%) | 2 (20) | 2 (20) | 1.000 |
| Hyperlipemia, n (%) | 4 (40) | 3 (30) | 1.000 |
| CHD, n (%) | 1 (10) | 2 (20) | 1.000 |
| CVD, n (%) | 3 (30) | 0 (0) | 0.211 |
| Current smoking, n (%) | 1 (10) | 0 (0) | 1.000 |
| Current drinking, n (%) | 3 (30) | 2 (20) | 1.000 |
| Current coffee drinking, n (%) | 3 (30) | 1 (10) | 0.582 |
| Current tea drinking, n (%) | 8 (80) | 6 (60) | 0.208 |
| Regular exercise | 8 (80) | 9 (90) | 1.000 |
| Milk for 250 ml /d , n (%) | 9 (90) | 9 (90) | 1.000 |
| Eating one egg /d , n (%) | 9 (90) | 10(100) | 1.000 |
| Fresh vegetables and fruits > 250g/d, n (%) | 8 (80) | 9 (90) | 1.000 |
| Grains and tubers, n (%) |  |  | 1.000 |
| <100g / meal | 2 (20) | 2 (20) |  |
| 100g-150g / meal | 8 (80) | 8 (80) |  |
| Unprocessed red meat, n (%) |  |  | 1.000 |
| <100g / d | 3 (30) | 4 (40) |  |
| 100g-150g / d | 7 (70) | 6 (60) |  |
| Unprocessed white meat, n (%) |  |  | 0.582 |
| <100g / d | 7 (70) | 9 (90) |  |
| 100-150g / d | 3 (30) | 1 (10) |  |
